# The Therapeutic Potential of Regulatory T Cells: Challenges and Opportunities

**DOI:** 10.3389/fimmu.2020.585819

**Published:** 2021-01-15

**Authors:** Fatemeh Bayati, Mahsa Mohammadi, Maryam Valadi, Saeid Jamshidi, Arron Munggela Foma, Ehsan Sharif-Paghaleh

**Affiliations:** ^1^Department of Immunology, School of Medicine, Tehran University of Medical Sciences, Tehran, Iran; ^2^Research & Development Department, Aryogen Pharmed, Karaj, Iran; ^3^Department of Stem Cells and Developmental Biology, Cell Science Research Center, Royan Institute for Stem Cell Biology and Technology, ACECR, Tehran, Iran; ^4^Department of Developmental Biology, University of Science and Culture, Tehran, Iran; ^5^Department of Imaging Chemistry and Biology, School of Biomedical Engineering and Imaging Sciences, Faculty of Life Sciences and Medicine, King’s College London, London, United Kingdom

**Keywords:** regulatory T cells, natural regulatory T cells, induced regulatory T cells, cancer, autoimmune disorders, transplantation, clinical trial

## Abstract

Regulatory T cells (Tregs) are an immunosuppressive subgroup of CD4^+^ T cells which are identified by the expression of forkhead box protein P3 (Foxp3). The modulation capacity of these immune cells holds an important role in both transplantation and the development of autoimmune diseases. These cells are the main mediators of self-tolerance and are essential for avoiding excessive immune reactions. Tregs play a key role in the induction of peripheral tolerance that can prevent autoimmunity, by protecting self-reactive lymphocytes from the immune reaction. In contrast to autoimmune responses, tumor cells exploit Tregs in order to prevent immune cell recognition and anti-tumor immune response during the carcinogenesis process. Recently, numerous studies have focused on unraveling the biological functions and principles of Tregs and their primary suppressive mechanisms. Due to the promising and outstanding results, Tregs have been widely investigated as an alternative tool in preventing graft rejection and treating autoimmune diseases. On the other hand, targeting Tregs for the purpose of improving cancer immunotherapy is being intensively evaluated as a desirable and effective method. The purpose of this review is to point out the characteristic function and therapeutic potential of Tregs in regulatory immune mechanisms in transplantation tolerance, autoimmune diseases, cancer therapy, and also to discuss that how the manipulation of these mechanisms may increase the therapeutic options.

## Introduction

Regulatory T cells (Tregs) are a specialized CD4^+^ subpopulation of lymphocytes with regulatory functions that suppress excessive and uncontrolled immune responses, which inhibit adaptive and innate immune cells such as conventional T cells, B cells, antigen-presenting cells (APCs), natural killer (NK) cells, and so forth. Several investigations have shown that Tregs play a significant role in the maintenance of immune homeostasis and self-tolerance (1, 2).

Organ transplantation is the gold standard therapy for end-stage organ failure. Although, the results of organ transplantation have been ameliorated in recent decades, chronic rejection and the side-effects of immunosuppressants are still an ongoing serious issue (3). None of the present immunosuppressive medications (in contrast to Tregs) have the potential to specifically suppress immune mechanisms. Various strategies are currently underway to avoid or minimize the use of immunosuppressive drugs. In this case, it may be possible that Tregs represent a promising solution to induce transplantation tolerance and control the immune response (4).

Autoimmune diseases as lifelong disorders are one of the major causes of mortality. The main etiology of autoimmune diseases is not fully understood; however, failure of immunological tolerance is a common cause of each autoimmune condition (5). Due to the discovery of involvement of Tregs in these patients (6), Treg-based cellular therapies are opening the door to new therapeutic options for autoimmunity (7).

Cancer is the second leading cause of morbidity and mortality worldwide. It is a well-acknowledged fact that cancer basically arises from uncontrolled growth and division of self-cells. Tregs play a pivotal role in tumor immune evasion by suppressing the immune responses of tumor-attacking immune cells (8). Treg-targeting antibody treatment through selective Treg depletion, suppression of Tregs function, and inhibition of Tregs migration to tumor site makes the cancer immunotherapy more effective (9).

In this review, we will summarize the role of Tregs in transplantation, autoimmunity, and cancer which can hopefully be used for developing clinical Treg-based therapies.

## Origin, Phenotype, and Markers

CD4^+^CD25^+^ Tregs constitute approximately 5–10% of total CD4^+^ T cells and about 1–3% of circulating CD4^+^ T cells in humans (10). These cells have effective roles in immune homeostasis preservation (maintenance) (11), immune tolerance, inhibition of autoimmune diseases as well as allergy development and in the control of anti-cancer immune responses (12). The expression of the transcription factor forkhead box P3 (Foxp3) and the high-affinity interleukin-2 receptor alpha chain (IL-2R*α* or CD25) are defining features of Tregs (13).

Tregs were primarily represented based on the anatomical site where they were differentiated (14):: Natural Tregs (nTregs) develop in the thymus and migrate to the periphery. Adaptive or induced Treg (iTreg) develop from conventional naive Foxp3^−^CD4^+^ T cells in the periphery. Both nTreg and iTregs constitute populations of peripheral Foxp3^+^Tregs (15, 16). However, with the extended knowledge on Treg biology, studies have indicated that CD4^+^ T cells can actually differentiate to various subsets *in vivo* (in both thymus and periphery regions) and *in vitro* (17). Therefore, the nomenclature is becoming to some extent, inexact and equivocal. For instance, distinction among different types of iTregs generated *in vitro* or *in vivo* is often confusing (18). Hence, in order to simplify Treg nomenclature, a list of recommendations were proposed at the third international conference on regulatory T cells and Th subsets and clinical application in human diseases (19):

1. ‘Thymus-derived Tregs (tTregs)’ should be employed instead of ‘natural T regulatory cells (nTregs)’.
2. ‘Peripherally-derived Tregs (pTregs: Foxp3^+^ Tregs that differentiate in the periphery)’ should be used rather than ‘induced or adaptive Tregs (iTregs or aTregs).
3. ‘*In vitro*-induced Tregs (iTregs)’ should be used to clearly distinguish between the Treg populations generated *in vivo versus in vitro*.

**Figure 1:**
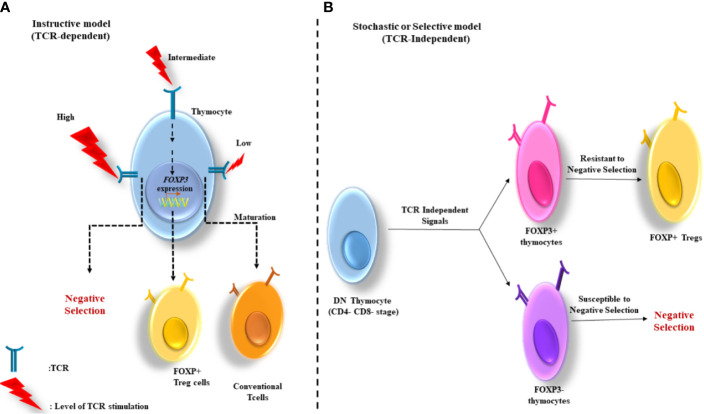
Two major models for thymus-derived Treg (tTreg) development. **(A)** *The instructive model:* Based on this model, the level of TCR stimulation determines the fate of thymocytes. The cells negative selection and maturation to conventional T cells are induced by the stimulation of TCR at high and low levels, respectively. Whereas, the intermediate stimulation of TCR contributes to *FoxP3* gene expression. **(B)** *The stochastic or selective model:* In this model the induction of *FoxP3* gene expression is independent from the strength of TCR signaling and occurs in a CD4^-^CD8^-^ double negative (DN) stage. Therefore, the development of two major groups of T cells (FoxP3^+^ and FoxP3^−^) occurs in the thymus. The FoxP3^+^ cells, with strong reactivity with self-antigens are high resistance to negative selection, and therefore develop into Tregs.

Moreover, the tTreg development in human remains unknown, so two major models have been suggested for thymic Treg (tTreg) development:

1. The instructive model: According to this model, the stimulation level of T cell receptor (TCR) determines the thymocyte fate. High and low levels of TCR stimulation induce the negative selection and maturation of the cells to conventional T cells, respectively. Whereas, intermediate TCR stimulation (among negative and positive selection) gives rise to *Foxp3* gene expression (**Figure 1A**) (20).

2. The stochastic or selective model: Unlike the instructive model, in this model the induction of *Foxp3* gene expression is independent of the strength of TCR signaling and occurs in the double negative (CD4^–^CD8^−^) stage. Thus, two groups of T cells develop in the thymus (Foxp3^+^ and Foxp3^−^). The Foxp3^+^ group whose TCRs react strongly with self-antigens are resistant to negative selection and consequently develop into Tregs (20) (**Figure 1B****)**.

On the other hand, pTregs can differentiate from Foxp3^−^ CD4^+^ T cells in secondary lymphoid organs to become Foxp3^+^ cells (21). These cells can also differentiate under sub-immunogenic conditions or in a non-inflammatory environment. Furthermore, pTreg differentiation can occur in a long-lasting (chronic) infection and inflammation as well as for the maintenance of gut homeostasis, and it can be generated in various forms of cancer similar to tTreg (22).

**Figure 2:**
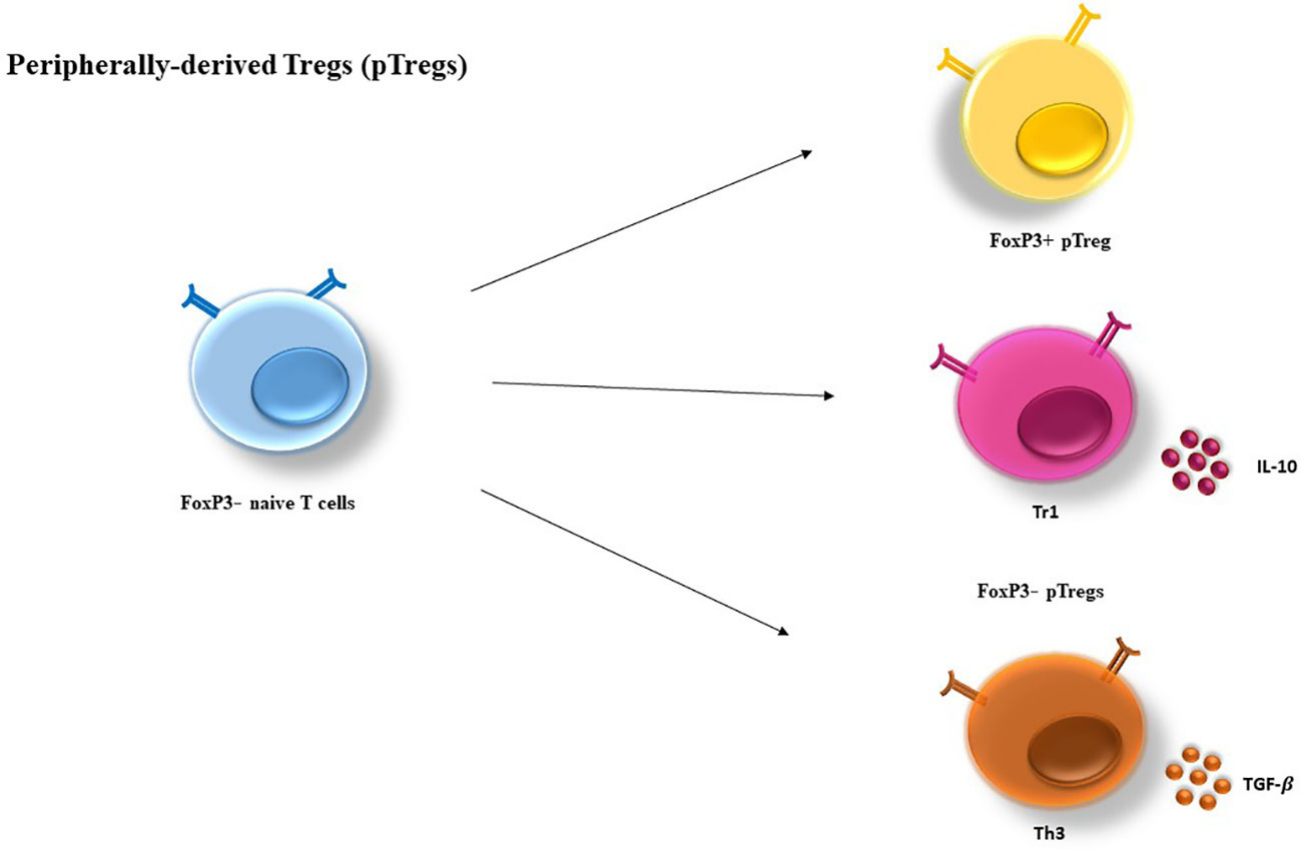
Peripherally-derived Treg (pTregs) development. pTregs can preferentially differentiate from FoxP3^−^ CD4^+^ T cells in the periphery and convert to FoxP3^+^ cells. pTreg differentiation can take place in a long-lasting infection, sub-immunogenic conditions and in the maintenance of gut homeostasis. In addition, two types of FoxP3^−^ have been defined as Tr1 and Th3, which are characterized by their cytokine profiles producing high levels of IL-10 and TGF-*β*, respectively. IL-10, Interleukin-10; TGF, transforming growth factor.

Moreover, two subsets of Foxp3^−^ Tregs have been discovered with suppressor functions: type 1 Treg (Tr1) and Th3 cells differentiate from peripheral naive CD4^+^ T cells (16, 23). As opposed to tTregs, Th3 and Tr1 suppressive properties are contact independent and is largely relying on cytokines including IL-10 and transforming growth factor-*β* (TGF-*β*) (24) (**Figure 2**).

It has been proved that TCR repertories of tTregs and pTregs are different: tTreg TCR repertoire is directed towards self-recognition and TCRs expressed in pTregs can identify non-self-antigen with high affinity (25).

While Foxp3 expression is regarded a reliable marker to recognize Tregs in mice (26), in certain circumstances it can be expressed by some conventional T cells especially in human (27). Alternatively, to recognize Tregs, all CD4^+^ T cells can be categorized based on the expression of CD45RA and Foxp3 (28): (a) naive/resting Tregs, characterized by Foxp3^lo^CD45RA^+^CD25^lo^ cells; (b) effector Tregs characterized by Foxp3^hi^CD45RA^-^CD25^hi^ cells; and (c) non-Tregs, characterized by Foxp3^lo^CD45RA^-^CD25^lo^ cells (29).

It is noteworthy that specific markers of tTreg and pTreg in human have not been detected to date (25). New study showed that a molecule called GPA33 is highly expressed in tTreg at both the mRNA and protein level (30). It has also been demonstrated that GPA33 expression was not observed on TGF-*β*-induced Tregs and also on iTregs. It is important to note that this receptor is expressed by some conventional T cells. As a consequence, GPA33 is not an individual cell marker by itself, as it applies to any other cell markers used to recognize the cells such as Foxp3 (31), Neuropilin (NRP-1) (32) and Helios (33). Although, following TCR-mediated activation, GPA33 no longer is expressed by conventional T cells, the expression of this molecule continues steadily on Tregs (30). Employing the combination of proper substitute markers is required to result in an optimal population for applications in Treg therapy (34).

## Suppressive Mechanisms

The first investigation which described tTreg development in the thymus (35) and its suppressive function (36) was demonstrated by Nishizuka and Gershon and their colleagues in 1969 and 1970, respectively. However, the suppressive function of Tregs was definitely demonstrated by Gershon and colleagues in 1972 (37).

It seems that tTregs exert their inhibitory function on the proliferation and function of effector T cells in a contact-dependent manner *via* receptors like CTLA-4 (Cytotoxic T lymphocyte antigen-4) and PD-1 (Programmed cell death protein 1). In contrast, pTregs mainly exert their inhibitory function *via* soluble factors such as TGF-*β*1 and IL-10 (16).

Moreover, Tregs exert their immunosuppressive effects on diverse T cell subsets such as CD4^+^/CD8^+^ T cells, dendritic cells (DCs), B cells, macrophages, mast cells, NK cells, and natural killer T (NKT) cells (38). These cells can also suppress multiple subsets of CD4^+^ T cells such as T helper 1 (Th1), Th2, Th17 (39, 40).

**Figure 3:**
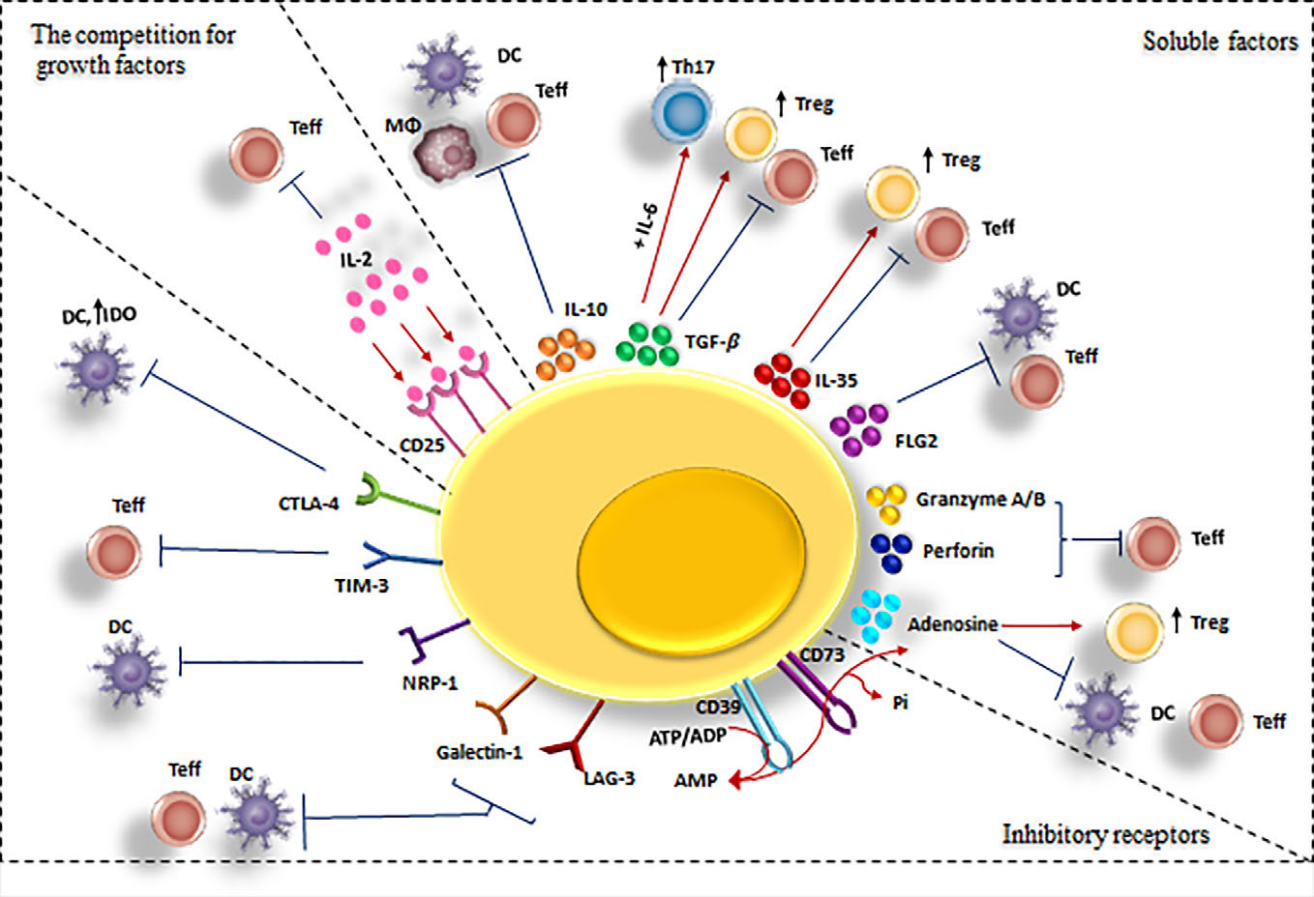
Cell-mediated suppression mechanisms of Tregs. A variety of molecular mechanisms might operate in a complementary fashion to contribute to Treg-mediated suppression. Tregs exert these suppressive effects on different cell types mainly via three mechanisms: 1) Producing soluble factors such as anti-inflammatory cytokines (IL-10, IL-35, and TGF-β), FLG-2, adenosine, granzyme and perforin. 2) Competing for growth factors: high-affinity CD25 receptors on Tregs and effector T cells compete for the relating ligands.3) Inhibitory receptors: Tregs have been observed to have a direct effect on target cells via interaction of CTLA-4, TIM-3, NRP-1, Gal-1 and LAG-3 and their ligands. CTLA-4, cytotoxic T lymphocyte-associated antigen-4; DC, dendritic cell; FLG-2, fibrinogen-like protein-2; GAL-1, Galectin-1; LAG3, lymphocyte activation gene 3; TGF, transforming growth factor; Treg , regulatory T cell; Teff, effector T cells; TIM-3, T cell immunoglobulin and mucin domain 3; Th17, T helper17; Nrp-1, Neuropilin; MQ, macrophage; IL-2, Interleukin-2; IL-6, Interleukin-6; IL-10, Interleukin-10; IL-35, Interleukin-35.

Generally, Tregs exert their regulatory effects in three ways: *via* soluble factors, inhibitory receptors, and the competition for growth factors (11, 41) (**Figure 3**). Specifically, they suppress CD4^+^ T cells directly *via* these mechanisms and indirectly by their inhibitory effects on APCs (38).

1. Soluble factors that mediate Tregs inhibitory effects are IL-10 (42), TGF-*β* (43), IL-35 (44), fibrinogen-like protein-2 (FLG-2) (45), granzyme A/B and perforin (46), and adenosine production by CD39/73 cleavage of ATP (47) (**Table 1**).

**Table 1:** Soluble factors that mediate Tregs inhibitory effects.

Soluble factors	Effects	References
**IL-10**	Suppressive effects on effector T cells and the production of IL-2, IFN-*γ*, IL-4, IL-5 and TNF-α Inhibition of APC maturation	(42)
**TGF-*β***	Role in pTreg differentiation and proliferation Th17 effector cells development (in presence of IL-6/21) Required for pTreg generation & maintenance of *Foxp3* expression in tTreg and pTreg Suppression of effector T cells Critical for the oral tolerance induction	(11, 48)
**IL-35**	Suppressing CD4^+^CD25^-^ effector cells proliferation Inhibition of Th17 cell polarization Stimulation of IL-10 generation Role in CD4^+^CD25^+^Treg expansion	(44, 49)
**FLG-2**	Apoptotic effects on effector T cells Inhibition of DC maturation	(11)
**Granzyme A/B and perforin**	Apoptotic effects on effector T cells	(11)
**Adenosine**	Cell cycle arrest in effector T cells by binding to the A2A receptor Down-regulation of NF-*κ*B activation & decreased release of proinflammatory cytokines and chemokines in effector T cells Prevention of maturation and decreased antigen presenting capability in DCs Promotion of Tregs expansion	(11, 50)

APC, antigen presenting cells; DC, dendritic cell; FLG-2, fibrinogen-like protein-2; TGF, transforming growth factor; Treg, regulatory T cell; Teff, effector T cells; Th17, T helper17; MQ, macrophage; IL-2, Interleukin-2; IL-4, Interleukin-4; IL-5, Interleukin-5; IL-10, Interleukin-10; IL-35, Interleukin-35.

*IL-10*: IL-10 is one of the most important anti-inflammatory cytokines. The activity of this dimeric cytokine is dependent on its interaction with its high-affinity receptor (IL-10R1) and subsequently with its low-affinity receptor (IL-10R2). With the formation of this ternary complex, signal transduction occurs and this cytokine can affect different cells expressing these receptors (51). By binding its receptor, IL-10 inhibits tyrosine phosphorylation in CD28 (the costimulatory molecule CD28 is involved in the interaction between effector cells and APCs), inhibiting PI3K/AKT activation, which in turn inhibits the signaling cascade leading to NF-*κ*B translocation (52). IL-10 demonstrates various biological activities notably immunosuppression, anti-inflammation, and immunomodulation. IL-10 can suppress the expression of major histocompatibility complex (MHC) class I in B and T cells and also in DCs, all of which drive inflammatory responses (53).

Transforming growth factor β (TGF-β): This pleiotropic cytokine has potent inflammatory and regulatory functions (54). By binding to TGF-*β* receptor II (TGF-*β*RII), it initiates the kinase activity of this receptor, leading to TGF-*β*RI activation. Following the effects of this activated receptor, Smad molecules translocate to the nucleus, resulting in the transcription of target genes (55). Also, the expression of GATA3, T-bet, signal transducer and activator of transcription 4 (STAT4), IFN-*γ* (interferon-*γ*), and granzyme-B genes, are suppressed by TGF-*β* which have essential roles in the differentiation and function of effector T cells (56–60).

Moreover, this cytokine also plays an important role in inflammation. For instance, Th17 differentiation from naive T cells is induced in the presence of TGF-*β* and IL-6 (61).

In addition, TGF-*β* is essential for naïve T cells survival and it increases the differentiation of pTregs in the presence of IL-2 and Retinoic Acid (RA) and supports maintenance of tTregs (62). In addition, TGF-*β* exerts its regulatory effects by suppressing innate immune system cells; for instance, dampening functions of DCs (antigen presentation) (54), down-regulation of NK cell function (63), inhibition of type I (proinflammatory phenotypes) and promotion of type II macrophage (MQ) and neutrophil development (64).

IL-35: This cytokine is a heterodimeric member of the IL-12 family (IL-12, IL-23, IL-27) (65). Several anti-inflammatory effects of IL-35 have been reported: induction of CD4^+^CD25^+^ Tregs proliferation, IL-10 and TGF-*β* secretion, suppression of CD4^+^CD25^−^ effector cells proliferation, and inhibition of Th17 differentiation (66, 67). Nonetheless, many research have indicated that IL-35 mediates immune suppression in mouse model but have limited effectiveness in humans (68, 69). In an experiments IL-35 was produced by Treg through strong stimulation (70).

FGL-2 (Fibrinogen-like protein-2): This protein is a member of the fibrinogen-related protein superfamily. In addition to its major role in thrombosis, this cytokine is also secreted by T cells and highly expressed by Tregs (71). FGL-2 has a direct impact upon the polarization of T cells toward Th2 cells and down-regulating Th1 and Th17 immune responses both *in vivo* and *in vitro* (71). Moreover, it is well established that FGL-2 plays a key role in inhibiting DCs maturation *via* mechanisms such as abrogating the expression of CD80 and MHC class II molecules (72). This protein exerts its effects by binding to inhibitory Fc*γ*RIIB receptors expressed on APCs such as DCs, endothelial, and B cells (45).

Granzyme A/B and perforin: Granzyme A/B, are a family of serine proteases released from lymphocyte cytoplasmic granules. Granzymes after entering target cells cleave caspases and activate these intracellular enzymes (73). Perforin is a 60–70 kDa (kilodalton) glycoprotein and similar to complement component 9 (C9) is a pore-forming protein (74). After polymerization of this protein on the membrane of target cells, passive and non-selective transportation of diverse molecules such as ions, water and enzymes occurred (75).

During the interaction between Tregs and effector cells, the released granzymes from secretory granules can enter the effector cells through perforin channels or in a mannose-6-phosphate receptor-dependent manner. As a result of the aforementioned, apoptosis is induced by caspase dependent or independent pathways in effector cells (53).

It has been demonstrated that 5–30% of CD4^+^Foxp3^+^Tregs expressed a high level of granzyme B in tumor environments (53). Tumor-residing Tregs are capable of inducing apoptosis in NK and CD8^+^ T cells in a granzyme B and perforin-dependent manner (76).

Furthermore, several studies in the context of transplantation have reported that granzyme B has an essential role in the tolerance induction of Tregs (77).

CD39/CD73/adenosine: ATP can function as a proinflammatory molecule through both stimulating innate immune responses and inducing the activation of effector T cells (78).

ATP can upregulate the expression of CD86 on DCs. CD86 is a costimulatory molecule which is expressed on APCs and is required for T cell activation and survival (78).

**Figure 4:**
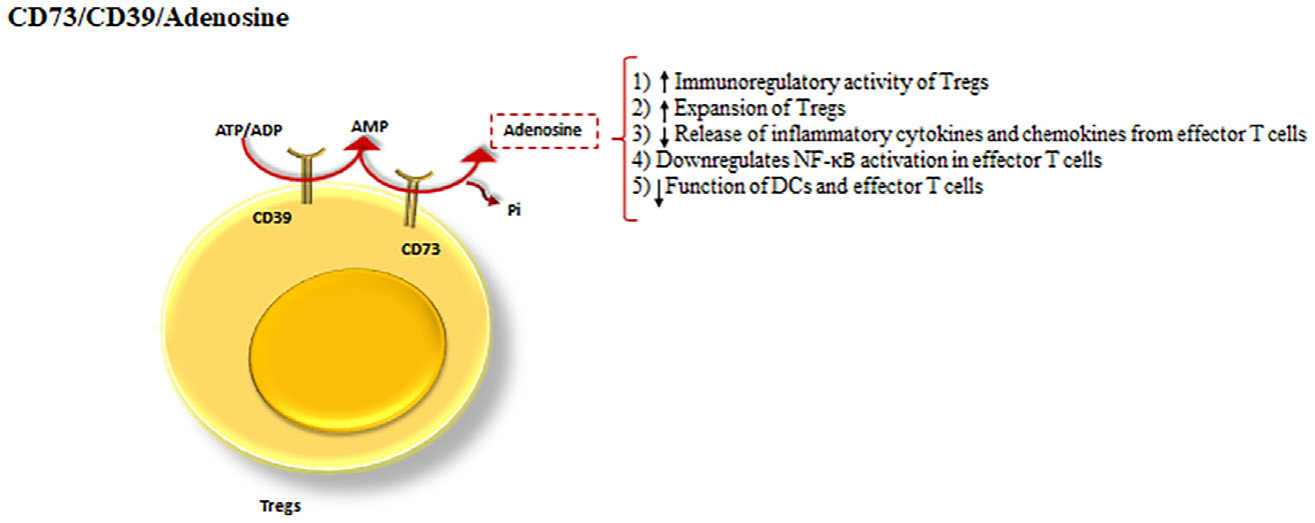
CD39 and CD73 cooperation as a Treg inhibitory mechanism. The cooperation between CD39 and CD73 as the powerful inhibitory mechanisms of Tregs. CD39, hydrolyses ATP to AMP and CD73 dephosphorylates the product of CD39, turning AMP into adenosine. Adenosine by means of the processes drives a shift from ATP-driven pro-inflammatory immune cell activity to an anti-inflammatory state.

CD39 and CD73 cooperatively shift ATP-driven pro-inflammatory immune cell activity towards an anti-inflammatory state, mediated by pericellular adenosine generation (79). Additionally, CD39 is an ectonucleotidase enzyme that hydrolyzes ATP or ADP to AMP (50) and CD73 dephosphorylates the CD39 product, AMP into adenosine (79) (**Figure 4**).

**Figure 5:**
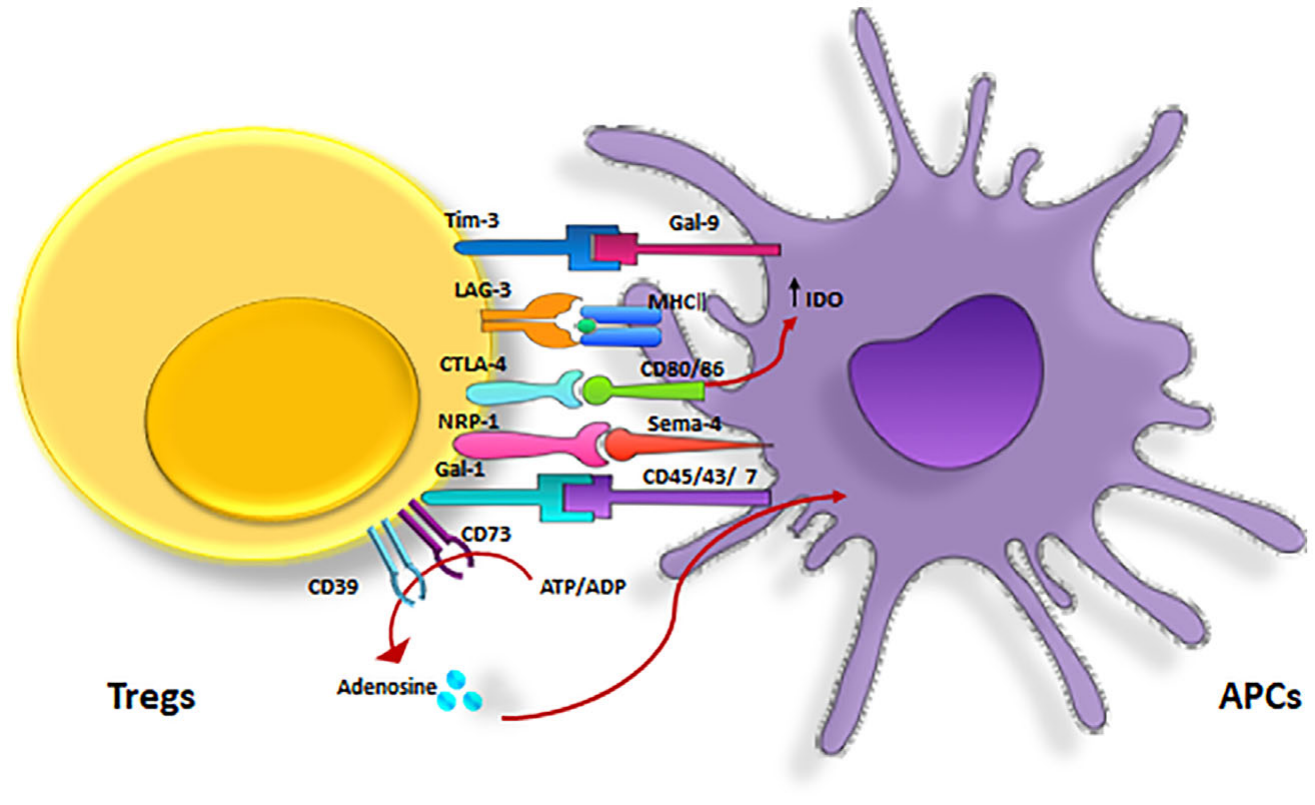
Inhibitory receptors expressed on Tregs. Treg inhibitory effects are mediated by several major receptors such as, CTLA-4, LAG-3, Tim-3, CD39/CD73, NRP-1 and Gal-3. CTLA-4, cytotoxic T lymphocyte-associated antigen-4; GAL-3, Galectin-3 ; LAG3, lymphocyte activation gene 3; TIM-3, T cell immunoglobulin and mucin domain 3; Nrp-1, Neuropilin.

2. Receptors that mediate Tregs inhibitory effects are CTLA-4 (Cytotoxic T lymphocyte antigen-4), Nrp-1, Galectin-1, LAG-3 (lymphocyte activating gene-3), and T cell immunoglobulin and mucin domain 3 (TIM-3) (80) (**Figure 5**, **Table 2**).

**Table 2:** Inhibitory receptors that mediate Tregs inhibitory effects.

Inhibitory receptors	Effects	References
**CTLA-4**	Blocking the subsequent increase of CD80 and CD86 expression and down-regulates the expression of CD80 and CD86 Decreasing the antigen presentation ability of DCs Promoting the secretion of suppressive factors (*i.e.* IDO) by DCs	(11, 38)
**Nrp-1**	Increasing interaction of Tregs with DCs Decreasing antigen presentation in DCs	(11, 81)
**Galectin-1**	Plays an important role in Treg-DC or Treg-T cell interactions Cell cycle arrest in effector T cells and DCs Inducing apoptosis in effector T cells and DCs Inhibit proinflammatory cytokines production	(11, 38, 82)
**LAG-3**	Preventing DC maturation Reducing DC antigen presentation capability Suppressing effector T cells	(11, 38)
**CD39**	Ectonucleotidase enzyme that hydrolyses ATP or ADP to AMP AMP Allows the Tregs to enter inflamed regions and allow the Tregs decreasing ATP-driven proinflammatory processes in different cell types, particularly DCs	(38, 83)
**CD73**	Turning AMP to adenosine Adenosine: Inhibits the functions of DCs as well as effector T cells Down-regulates NF-*κ*B activation in effector T cells Reducing the release of many proinflammatory cytokines and chemokines from effector T cells Increasing expansion of Tregs Increasing immunoregulatory activity of Tregs	(38, 47, 50, 84)

CTLA-4, cytotoxic T lymphocyte-associated antigen-4; LAG3, lymphocyte activation gene 3; Nrp-1, Neuropilin.

CTLA-4 (Cytotoxic T lymphocyte antigen-4; CD152): This structural homolog of CD28 is essential for the tolerance induction and homeostasis in T cells (85). Similarly, CTLA-4 and CD28 have the same ligand, CD80 (B7.1) and CD86 (B7.2) on APCs. The main role of this inhibitory molecule is to reduce the costimulatory signaling, mainly by the following ways:

competing with CD28 binding to CD80 and CD86, transmitting inhibitory signals through the induction of cell cycle arrest, inhibiting the secretion of IL-2 (86), down-regulating the expression of CD80 and CD86 ligands (65), and finally limiting the time exposure between T cells and APCs (86).

This protein is constitutively expressed on Tregs but can also be expressed on conventional T cells after their activation. Notably, CTLA-4 has an essential role in suppressing self-reactive T cells, *in vivo* (87).

Nrp-1(Neuropilin): Nrp-1 is a receptor belonging to the class III semaphorins and acts as a co-receptor for vascular endothelial growth factors (VEGFs) (38). In addition, Nrp-1 has an important role in initiating primary immune responses by mediating the immunological synapse formation between T cells and DCs (88). Nrp-1 is preferentially expressed on Tregs but not on naïve Th cells. This receptor promotes Treg interaction with immature DCs (iDCs) and limits the accessibility of effector T cells to APCs (81).

This receptor is constitutively expressed on murine Tregs and can be considered as an advantageous murine Treg surface marker (89). Although, unlike mice Tregs, Nrp-1 expression on both human conventional T cells and Tregs is consistently low and cannot be regarded as a human Treg marker (90).

In addition, a decreased expression level of human Nrp-expressing Tregs is observed in biopsies from rejected renal graft (91). It is also shown that Nrp-1 contributes to intratumoral Treg stability, and the high percentage of Nrp1^+^ Tregs in tumor environments correlates with poor prognosis in both human melanoma and head and neck squamous cell carcinoma (92). Altogether, it seems that similar to murine Tregs, Nrp-1 has a resembling immunosuppressive function in human Tregs (88). *Galectin-1*: Galectin-1 is a member of *β*-galactoside-binding proteins. This protein is overexpressed on Tregs (human and mouse) and enhances its expression after Treg activation. The blockade of this protein inhibits the regulatory effects of Tregs. So, it seems that galectin-1 has an essential role in the regulatory function of Tregs (82).

Galectin-1 increases the apoptosis and cell-cycle arrest in activated T cells (93, 94). Moreover, this protein by binding to cell surface glycoproteins such as CD45, CD43, CD7, CD3, and CD2 prevents proinflammatory cytokines IL-2 and IFN-*γ* secretion and stimulates the secretion of the anti-inflammatory cytokine IL-10. Galectin-1 induces cell-cycle arrest and apoptosis in activated immune cells (94, 95). However, it is not yet clear whether galectin-1 exerts its effects as a secreted homodimer cytokine or as a cell surface molecule *via* cell-cell contact (38).

Lymphocyte Activation Gene 3 (LAG3, CD223): This transmembrane protein is a CD4 homolog (96). It binds to MHC class II molecules with a higher affinity than CD4 molecules, abrogating TCR-mediated activation responses in CD4^+^ cells (97).

Engagement of MHC II with LAG-3during Treg-DC interactions results in suppression of maturation and immunostimulatory capacity of DCs (98). Additionally, It has been demonstrated by Maruhashi et al. that LAG-3 selectively inhibits the T cells’ activation and, hypothetically other cells expressing LAG-3, involving CD8^+^ T cells, through binding to MHC class II/peptide complexes with stable structural conformation (99).

T cell immunoglobulin and mucin domain 3 (TIM-3): TIM-3, a negative regulator of immune tolerance, is a member of the TIM family (100). This receptor was first discovered as a molecule exclusively expressed on Th1 and CD8^+^ T cytotoxic 1 (Tc1) T cells. In recent years, this molecule has also been detected on Tregs and other cells such as DCs, NK cells, and monocytes (101).

A number of TIM-3 ligands have been discovered, namely Galectin-9, carcinoembryonic antigen-related cell adhesion molecule-1 (Ceacam-1), phosphatidylserine (PtdSer), and high mobility group protein B1 (HMGB1) (101–103). TIM-3 is constitutively expressed on mice tTreg (104) but not on human peripheral blood Tregs (105).

Moreover, less is understood about TIM-3 immunosuppressive functions in Tregs (101). In a study conducted by Gautron et al. it was indicated that TIM-3^+^ Tregs efficiently suppress effector Th17 cells, in contrast to TIM-3^−^ Tregs. In comparison with Tim-3^−^ Tregs, Tim-3^+^ Tregs reveal strong expression of IL-10 and CD39, and also other immune checkpoint receptors such as CTLA-4, LAG-3, and PD-1 (104). This suggests that targeting TIM-3^+^ Tregs could be a potential therapeutic strategy in cancer treatment (106).

3. The competition for growth factors (mainly IL-2) (48):

IL-2 is mainly produced by activated CD4^+^ T cells and plays an essential role in T cell activation (107). This cytokine is also produced by other cells such as, naive CD8^+^ T cells, DCs and thymic cells (108).

IL-2 receptor (IL-2R) is composed of IL-2R*α* (CD25), IL-2R*β* (CD122) and common *γ*-chain (CD132) (108). Tregs constitutively express CD25, in contrast to conventional T cells and compete for IL-2 with effector T cells (107). IL-2 deprivation prevents the proliferation of effector T cells and subsequently induces their apoptosis (109). However, IL-2 capture plays a negligible role for controlling CD4^+^ T cells; it is crucial for limiting CD8^+^ T cell activation (110). In addition, IL-2 is also essential for Treg homeostasis, development, stability, and function (111) and their efficient suppressing functions (112, 113). As opposed to developing Tregs which require IL-2 for Foxp3 expression, mature Tregs rely on persistent IL-2 signaling to sustain survival and inhibitory function (114). In a research by Shi et al. indicated that Mst1–Mst2 act as a vital regulator of IL-2–STAT5 signaling in Tregs through reinforcing IL-2R–STAT5 signal and enhancing access to low dose IL-2 to strengthen the lineage stability. From this standpoint, low-dose IL-2 therapy is proved to be optimistic approach in treating autoimmune diseases (115).

## Role in Transplantation

In 1975 Kilshaw et al. indicated the suppressing role of T cells in decreasing skin allograft rejection in mice (116). However, Hall et al. demonstrated the direct role of CD4^+^CD25^+^ T cells in increasing allograft survival in 1990. This study showed that CD4^+^CD25^+^ T cells can prolong cardiac allografts in rat models (117). Finally, in 1993, Qin et al. showed that CD4^+^ T cells from tolerant mice induced tolerance in naïve CD4^+^ T cells. Therefore, graft rejection did not occur even without using immune suppressive drugs (112).

The attained evidence on the role of Tregs in increasing transplantation tolerance (112) highlighted the importance of Tregs in the transplantation field. Furthermore, studies have shown that T cells respond to non-self proteins (116). Immune responses against allotransplantation result in the activation of different immune cells mainly T cells and macrophages which play an important role in graft rejection.

Graft antigens are presented to T cells by two major pathways: 1. The direct pathway: in this pathway, recipient T cells recognize donor APCs’ MHC, directly. This pathway has an important role immediately after transplantation. In fact, graft resident APCs migrate to lymphoid tissues and present their surface MHCs to resident T cells in these tissues. Finally, alloreactive T cells are activated. 2. The indirect pathway: in this pathway, processed MHCs derived from donor graft presented on recipient APCs to T cells and activate alloreactive T cells. Because of the short life span of donor APCs, the indirect pathway is more important in alloreactive T cells activation (118).

Anyhow, both tTreg and pTreg have the capability to inhibit both innate and adaptive immune cells. In allograft rejection, mainly conventional CD4^+^ and/or CD8^+^ T cells play an important role. These cells can recognize alloantigens that are presented from direct or indirect pathways and initiate alloreaction leading to allograft inflammation and graft rejection (119). Many studies have demonstrated the important role of Tregs in increasing allograft survival and induction of allograft tolerance. For example, Anderson et al. indicated that in murine allograft models, the depletion of CD4^+^CD25^+^ T cells from allografts, increased chronic allograft rejection and infusion of donor Tregs, inhibiting chronic graft versus host diseases (GvHD) (120). Overall, it has been demonstrated that Treg induction has a critical role in the tolerance up keeping in allograft transplantation (118). In order to enhance suppression *in vitro*, the ratio of CD4^+^CD25^+^ Tregs to effectors is estimated to be 1 to 3 (121). Keeping this in mind, infusion of Tregs that are expanded *ex vivo*, can be a promising way to treat allograft rejection (14).

### *Ex Vivo* Expansion Strategies of Tregs

#### *Ex Vivo* Expansion of Polyclonal Tregs

The purification of CD4^+^CD25^hi^CD127^low/−^ tTregs are performed using magnetic enrichment or fluorescence associated cell sorting (FACS). Highly purified tTregs are stimulated with coated anti-CD3/CD28 antibodies supplemented with a high amount of IL-2 (200–1,000 IU/ml) and in some cases rapamycin (100 ng/ml or 100 nM). Activated Tregs are then expanded *ex vivo* for several days to be prepared for infusion after checking the quality (122). In 2009, Trzonkowski et al. conducted the first clinical trial of treating patients with GvHD, using *ex vivo* expanded donor CD4^+^CD25^+^CD127^−^ Tregs. The results showed that one patient with chronic GvHD (2 years post bone marrow transplantation) was successfully withdrawn from immunosuppressive drugs without recurrence of GvHD after receiving the therapeutic Tregs. However, the second patient with acute GvHD (at one-month post-transplantation), died after Treg therapy despite temporary improvement (123). In another study (2011), Brunstein et al. showed that infusion of *ex vivo* expanded Tregs isolated from the umbilical cord, could reduce the incidence of grades II–IV of acute GvHD. However, no significant difference was observed in the overall incidence of GvHD in patients and control groups (124).

The investigators at the University of California (San Francisco) performed a phase I, open-label pilot study called TASK in 2017. The test was planned to determine the feasibility of Treg isolation, expansion and infusion in three kidney graft recipients using tacrolimus, mycophenolate mofetil, and corticosteroids with subclinical inflammation. This study has labeled expanded Tregs with deuterium using a medium containing deuterated glucose for tracking the autologous Tregs following infusion. No infusion reactions or severe therapy-related adverse events have been reported following the administration of a single infusion of virtually 320 × 10^6^ expanded Tregs in all recipients. The persistence pattern of infused cells was similar to what was observed in non-immunosuppressed type 1 diabetes (T1D) patients. Infused Tregs, which were well tolerated without any report of short-term toxicities (cytokine release and infusion reactions), or infectious complications, reached the peak from 2 to 8% of circulating Tregs in the first week. In all recipients, the deuterium signals maintained detectability in the first month of post-infusion and reduced close to detection limit of 0.2% at 3 months after infusion. Owing to the low number of participants it is not feasible to draw any solid conclusion concerning the efficacy of Tregs for diminishing graft inflammation. However, considering the feasibility and safety outcomes have paved the way to design a full-scale protocol of Clinical Trials in Organ Transplantation-21 (CTOT-21, NCT02088931) to determine the efficacy of infused polyclonal Tregs *versus* donor alloantigen-reactive Tregs in a randomized controlled trial (NCT02711826) (125).

The researchers at Northwestern University (Chicago, USA) conducted the Treg Adoptive Cell Therapy (TRACT) trial in which nine patients, who had undergone renal transplantation from living donors, received an escalating dose of *ex vivo* expanded polyclonal Tregs (500–5,000 × 10^6^). Treg therapy with varied doses have been reported safe with no record of adverse infusion-related side effects, infections or rejection events up to two years after transplantation in patients. This study offers the required data to promote Treg cell therapy to phase II efficacy trials (126).

An open-label, dose escalation, phase I clinical trial study was conducted to evaluate the safety, applicability, and biological activity of autologous polyclonal Treg therapy in adult cadaver liver graft recipients. Three subjects were administered 1 × 10^6^ Tregs/kg 4 months after transplantation and six recipients were given 4.5 × 10^6^ Tregs/kg 333–505 days after transplantation. Thymoglobulin, methylprednisolone, low dose tacrolimus, and rapamycin were given to the recipients. These Tregs demonstrated a favorable safety profile; however, only an individual showed an infusion reaction. The circulating Treg level swiftly elevated the following infusion and remained higher than pre-infusion for more than one month. In those patients who received 4.5 × 10^6^ Tregs/kg, the T responses against donor-type cells reduced moderately without any observable changes in T cell responses against third party alloantigens or the cytomegalovirus. The reason why such an effect was not observed with administration of 1 × 10^6^/kg Tregs may be associated with a potential causal and dose–effect relationship, while the influence of thymoglobulin-induced lymphopenia should be taken into account, which merely appeared in the low-dose Treg cohort. Some challenges were associated with the clinical protocol, and its applicability was dependent on the delay in patient recruitment and cell infusion at least 6 months post-transplant. Prospective investigations should optimize this approach alone or in combination with lymphodepletive therapies to attenuate or even wean off immunosuppressive drugs following liver transplantation (127).

Sawitzki et al, have recently published primary results of The ONE study, which is a multicentric international phase 1/2A study, to test the feasibility, safety, and efficacy of regulatory cell-based medicinal products (CBMPs) in adult renal transplantation recipients from living donors. The reference group along with the six different cell-based trials was conducted, in which recipients received one of six CBMPs involving Tregs, DCs, or macrophages. To date, none of the mentioned trials have included a multicenter or another comparator group to evaluate the results. The reference group trial was a standard-of-care group that received basiliximab, reduced steroids, mycophenolate mofetil, and tacrolimus. The Treg therapy cohort was not given basiliximab due to its potentiality to suppress infused Tregs. The results from host immune cells in the cell therapy group trials revealed lower infections and inflammatory responses with lower requirements of immunosuppressive therapy over a 60-week follow-up period in comparison with the reference group trial administered standard-of-care immunosuppression (128).

Some important issues need to be considered. Each of the six patient groups in all centers either before or after transplantation received a single infusion of one distinct cell type with different dose levels coupled with uniform triple drug immunosuppression. However, T cell products were administrated in post-kidney transplant; the infusion doses were different. Unlike the trial group, the recipients in the reference group received basiliximab induction, and after week 2 the dose of mycophenolate mofetil was reduced. Accordingly, the reference group trial could not be a real control group. In the cell therapy group trials, the lower incidence of infections may manifest the lower general immunosuppression. Another drawback is that the follow-up period was not long enough to evaluate the clinical endpoints, such as drug associated effects, which required over 1 year to be observed (129).

These data do not determine the most effective regulatory cell therapy regimen. More detailed reporting, thus, is required which can be provided by prospective individual cases based on the feasibility, safety aspects, and effects of each specific cell therapy product.

In the phase I clinical trial as a part of The ONE study, which was done at two centers in the U.K., patients were divided into Treg therapy cohort in which 1–10 × 10^6^ Treg per kg at Day +5 after transplantation given to 12 kidney transplant recipients to induce immunosuppression, and reference cohort in which 19 patients received standard immunosuppression. The results demonstrated that patients and graft survival was 100% and acute rejection-free survival rates were reported to be 100% in Treg therapy compared to 78.9% in the reference cohort after 48 months of post-transplant. Successful withdrawal of mycophenolate mofetil was observed in four patients in the Treg therapy cohort and remained on tacrolimus monotherapy. Treg infusion is associated with an enduring dose-based elevation in peripheral blood Tregs along with growing marginal zone B cell (IL-10-producing B cells) numbers (130).

In another phase 1 clinical trial as part of the ONE study, which was conducted in Berlin, Germany, the patients received polyclonal Treg products 7 days following the renal graft as a single intravenous dose of 0.5, 1.0, or 2.5–3.0 × 10^6^ cells/kg with ensuing gradual decrease of triple immunosuppression to low dose tacrolimus monotherapy up to week 48. In all of the three Treg dose escalation groups no dose-limiting toxicity has been reported. The Treg and reference groups revealed 100% three-year transplantation survival and similar clinical and safety profiles. Although stable monotherapy immunosuppression was obtained in 73% of subjects receiving Tregs, the reference group underwent standard dual or triple-drug immunosuppression. The Treg group demonstrated a little higher rate of the marginal zone B cell subset in the circulation at the 60-week duration of the study which is in line with the result of the study conducted in the UK. In the Treg group less conventional T cell activation, NK cell maturation, and down-regulation of the rejection-related gene were observed. The Treg infusion stimulated simply a short-lived increase in Tregs. The Treg homing to the inﬂamed transplantation or lack of long-term engraftment may account for the reduction in circulating Tregs following four weeks. The number of patients enrolled was inadequate, which can restrict the power of statistical analyses (131).

A randomized study called TWO study has been already designed on the basis of ONE study by the UK group with their polyclonal Treg cell product (ISRCTN11038572), and other ONE study collaborators performing the trials transplant recipients with cell products employed in the ONE study (NCT03577431 and NCT02188719).

None of the trials to date have addressed the homing and long-term viability of adoptively transferred Tregs. Their inability to persist in high numbers in the circulation may reflect the increased rate of apoptosis associated with low IL-2 availability or preferential migration into peripheral tissues. This challenge may be addressed by long-term tracking the injected cells *via* a novel non-invasive imaging technology in the future (127).

However, most of polyclonal Treg-based clinical trials in solid organ transplantation (mostly kidney and liver transplantation) are still in progress (4) (**Supplementary Table 1**). All clinical trial data published about Treg therapy in transplantation have confirmed Tregs’ safety (14) and tolerability even in a high dose infusion (3). However, some questions arise in the matter of the sources of Treg, isolation method, dose and timing of infusion, optimal immunosuppressive regimen, and cell fate post-infusion. It remains to be seen whether cell-based therapy with Treg, as a single potent agent for immunomodulatory, has the potential to induce immunosuppressive-free immune tolerance or not (125). So, it seems that more research must be done in this field in order to discover the efficacy in the treatment of transplant patients regarding the derived outcome from the ongoing clinical trials (3) (**Supplementary Table 1**). Despite the limited but promising success of polyclonal Tregs, infusion required a large number of cells, and the threat of non-specific immunosuppression is possible (126). To successfully deal with these restrictions, both required high cell numbers as well as the off-target specificity of polyclonal Tregs, an enriched population of alloantigen-specific Tregs could be used (127).

#### *Ex Vivo* Expansion of Alloantigen-Specific Tregs

In order to activate purified tTregs, donor APC or artificial APCs (K562 cell-based artificial APCs) pulsed with given antigen in presence of high IL-2 are used. Cell expansion is continued for several days followed by the evaluation of infusion post-quality (128). Donor APC is a vital reagent for generating alloantigen-specific Tregs. These APCs have been isolated solely from peripheral blood mononuclear cells (PBMCs) (129) or in conjunction with FACS sorting (130), DCs (131), and B cells (132, 133). Using B cells has advantages over DCs due to their comparative abundance and ease of expansion (133). Naïve B cells would fail to induce expansion of Tregs unless anti-CD28 agonist antibodies were added (134). Preliminary B cell expansion and activation steps are vitally important in using B cell for Treg allostimulation. Since B cell proliferation requires a costimulatory signal from CD40/CD40L, CD40L-expressing fibroblasts have been used as feeder cells for B cell expansion (127). As compliance with Good Manufacturing Process (GMP) in this method is mandatory for donor material to be collected, it is considered a limitation for this approach. To address this challenge, banked B cell application has been suggested (135); however, human leukocyte antigen (HLA)-donor/recipient may not be all covered by this bank (136). Additionally, Putnam et al. developed a manufacturing process using CD40L-activated allogeneic B cells to selectively expand alloantigen specific Tregs in human in short-term cultures using GMP-compliant reagents followed by polyclonal restimulation to multiply yield (133). Yet, this protocol may potentially cause cellular contamination of the final cell product. In a recently developed method (UltraCD40L) four trimers of CD40L are connected, and activated B cells can be significantly expanded independently of feeder cells which made this approach more clinically acceptable (128).

Considering that no scientific research has been conducted to directly compare expansion utilizing stimulatory cell populations as an alternative from the same donor, it is still inconclusive which approach would be the most effective for generating alloantigen specific Tregs (127). In 2016, Todo, et al. reported the use of alloantigen specific Tregs generated in living donor liver transplantation (137). In this study, the recipient lymphocytes were cultured with irradiated donor cells in presence of anti-CD80/86 monoclonal antibodies (mAbs) for 2 weeks. Immunosuppressive agents were diminished from 6 months followed by a reduction every 3 months, and completely stopped within 18 months. No major adverse effects were caused by these infused cells. At the moment, all patients maintain normal graft function and histology, seven of whom have successfully achieved weaning and completed cessation of immunosuppressive agents. Currently, they have been remained drug-free during 16–33 months, in whom four have been drug-free beyond 24 months. Although three patients with autoimmune liver disorder were diagnosed to develop mild rejection while weaning, then such patients were treated with conventional low-dose immunotherapy. However, there are some problems associated with the feasibility of this pilot study: this study involves a few numbers of patients with a limited follow-up period. Compared to deceased donor liver transplant, living donor liver transplant offers a more optimal immune status in inducing tolerance because of its short ischemia time and relative HLA-compatible. Ongoing studies regarding clinical trials of alloantigen-specific Tregs in the early phase of the solid organ transplantation setting are being conducted, and their results will be reported in years to come (**Supplementary Table 1**).

#### Chimeric Antigen Receptor Tregs

At present, by application of CARs, activating recipient Tregs with donor-derived APCs is no longer needed (138). CARs are artificial receptors comprised of an extracellular antigen-binding domain and an intracellular signaling domain; the cytoplasmic tail of CD28 and CD3*ζ* are fused together to propagate both TCR and costimulatory signals in a single receptor (139).

In principle, allografts’ HLA that is not expressed by the recipient can be a potential target for CAR to direct Treg specificity for organ transplantation. HLA-A is particularly highly dominant (>40%) in white organ donors (55, 56). Additionally, HLA-A mismatching is commonly connected with the poor grafting result after transplantation (126). Some investigators have engineered anti-HLA-A2 CARs (A2-CARs) for Tregs, and also human A2-CAR-Tregs were assessed both *in vitro* and *in vivo* (139–143). All studies demonstrated the *in vivo* efficacy of CAR-Tregs in the prevention of human against mouse *i.e.*, xenogeneic GvHD, or in controlling HLA-A2^+^ human skin grafts rejection mediated by alloimmune responses (144).

## Role in Autoimmunity

Sakaguchi et al. demonstrated that the depletion of CD4^+^CD25^+^ Tregs from mice led to autoimmune diseases in athymic mice. This suggests an important role of these cells in the inhibition of autoimmune diseases (145). Moreover, Tregs play an essential role in immune homeostasis and immune response regulation. So, disturbance in function, inadequate number of Tregs, and the resistance of effector cells to immune regulatory mechanisms of Tregs, can lead to autoimmune diseases (146).

In psoriasis, it is shown that the number of Tregs in the peripheral blood of psoriatic patients decreases (11). In addition, it is demonstrated that CD4^+^CD25^hi^ Tregs from patients with autoimmune diseases such as, Multiple Sclerosis (MS), Polyglandular syndrome type II, Myasthenia gravis or Rheumatoid Arthritis (RA) have impaired functions compared to Tregs from healthy individuals (147).

It has also been shown that effector T cells from autoimmune patients are resistant to the suppressive effects of Tregs (48, 106). For instance, CD4^+^CD25^−^ T cells from RA patients are resistant to inhibition by Tregs, in comparison to CD4^+^CD25^−^ T cells from healthy individuals (148). Therefore, it seems that the altered function and number of Tregs and effector T cells may play an indispensable role in autoimmune diseases (11).

Foxp3, a master marker of CD4^+^ Tregs, is a transcription factor that is encoded by the *Foxp3* gene. This transcription factor is necessary for Treg development, proliferation and its suppressive function (149). In Foxp3 deficient Tregs, the expression of some gene hallmarks such as *ctla4, il10* is reduced. However, other signature genes of effector T cells like *ifng, tnfα, il4, and il17* are obtained (150).

Furthermore, “loss of function” mutations at the *Foxp3* gene locus can lead to a Treg related autoimmune disease referred to as immune-dysregulation polyendocrinopathy enteropathy X-linked inheritance syndrome (IPEX) (151). Also, in other autoimmune diseases gene polymorphism at *Foxp3* locus has been reported. For instance, *Foxp3* polymorphisms in promoter, exon, intron or Poly A region of *Foxp3* gene locus have been detected in rheumatoid arthritis (RA), systemic lupus erythematosus (SLE), type 1 diabetes (T1D) and even in IPEX itself (150). So, understanding the molecular relationship between Foxp3 and autoimmune diseases can help us for the treatment of Treg-associated autoimmune diseases (150).

As mentioned, Tregs play a pivotal role in immune homeostasis and tolerance. Consequently, Treg-targeted therapies in a direct or indirect manner have been developed to treat autoimmune conditions (152).

### *In Vivo* Induction and Expansion of Tregs

In order to ameliorate autoimmunity, new drug targets have been based on molecules to enhance the *in vivo* induction and Treg expansion (152). A wide range of compounds have been proposed which affect Treg numbers and function indirectly including IL-2 (153), anti-CD3 (154), and Rapamycin (155). Defective IL-2 signaling in Tregs stems from the deficiency of IL-2 or IL-2R subunits CD25 and CD122, which adversely affect Treg survival resulting in autoimmunity. Since trimeric IL-2Rs have high affinity to IL-2, even low dose IL-2 can reduce inflammation by expanding Tregs (156). Indeed, the clinical trials of low-dose-IL-2 treatment has been examined in T1D, SLE, GvHD and other disorders (153).

Patients with GvHD that enrolled in a Phase I clinical study demonstrated a clinical response (157). Both elevated Treg counts and an increased in NK cells, which also express CD25, were seen in these patients. There was a correlation between administration and preferential sustained Treg expansion *in vivo* and suppression of the manifestation of chronic GvHD. However, IL-2 in proportion to its dose can promote the activation of CD8^+^ T cells and eosinophils and elevate other destructive leukocytes including NK cells (158).

It has been demonstrated that mucosal (oral or nasal) administration of CD3 mAb could induce autoimmune suppression in animal model of encephalomyelitis (159), collagen-induced arthritis (160), systemic lupus erythematosus (161), and diabetes (162).

In mice, orally administrated anti-CD3 antibody is immediately absorbed by the gut-associated lymphoid tissue (GALT), inducing CD4^+^CD25^−^LAP^+^ Tregs in the mesenteric lymph nodes which function to inhibit experimental autoimmune encephalomyelitis (EAE) and diabetes in a TGF-*β* dependent manner (163, 164).

Following the oral administration of OKT3 (anti-CD3 antibody), a reduced production of IL-17 and IFN-*γ*, and an increased expression of Treg markers (Foxp3, CTLA4, TGF*β*) were observed (165).

In the first study of six patients with moderate-to-severe ulcerative colitis who received oral anti-CD3 antibody, it was shown that it was well tolerated by all subjects. Nonetheless, it was not correlated with a change in Treg-associated molecule expression, namely Foxp3 and CTLA-4 (166). Currently, Foralumab (28F11-AE; NI-0401) is the only fully human anti-CD3 mAb (167). The entirely human origin mitigates the side effects that have been formerly associated with other humanized anti-CD3 mAb. The clinical trials of nasal and oral administration of Foralumab are being evaluated for patients with progressive MS and inflammatory bowel disease respectively (168).

The immunosuppressive drug rapamycin is commonly used in humans for preventing organ transplant rejection. Rapamycin permits the expansion of CD4^+^CD25^+^Foxp3^+^ Tregs in both murine and humans (169, 170). The Treg function in patients with T1D and Treg expansion in kidney transplant recipients can be promoted with rapamycin therapy (171). Additionally, this treatment improves clinical, histological, and immunological features in patients with IPEX syndrome, favoring its preferential use to restore their Treg function (169). Rapamycin is available for administration as an oral solution and in tablet form. However, the efficacy of rapamycin was demonstrated in patients with diverse pathological status, and its potency has significantly reduced due to the poor oral bioavailability, and the high free-plasma rapamycin sequestration into erythrocytes. The immunosuppressive potency of rapamycin can cause increased susceptibility of the host to viruses, infections, and even cancer (172).

### Treg Therapy

Treg therapy restores the dominant immune tolerance presumably by directly increasing the level of Tregs, giving rise to amplify immune suppression (152).

#### Polyclonal Treg Therapy

Polyclonal Treg administration is utilizing autologous *ex vivo* expanded Tregs for the restoration of immune tolerance in patients with autoimmune diseases. Some clinical trials employing polyclonal Treg therapy to treat autoimmune conditions have been accomplished or are in progress (**Table 3**). The first experience of Treg therapy in a patient with SLE has shown an increase in the level of activated Tregs in the inflamed skin. Accumulation of Tregs in the skin was along with severe impairment of the IFN-γ pathway and switch from Th1 to Th17 responses (173). It should be noted that this trial considered only one individual patient.

**Table 3:** Ongoing Treg-based clinical trials in autoimmunity.

Study ID	Phase	Intervention	Source	Dose	Drugs	Condition	Status	Location
**NCT03011021**	I/II	UCB-polyclonal Treg	Umbilical cord blood	1-5x10^6^/kg	Insulin Liraglutide	T1DM	Recruiting	Hunan, China
**NCT03239470**	I	Autologous polyclonal Tregs	NA	Cohort 1: 1x10^8^ cells Cohort 2: 2.5x10^8^ cells	NA	Pemphigus	Recruiting	Kentucky, United States
**NCT02691247**	II	Autologous polyclonal Tregs	NA	NA	NA	T1DM	Active, not recruiting	United States
**NCT02704338**	I/II	Autologous polyclonal Tregs	Peripheral blood	10–20x10^6^ cells/kg	NA	Autoimmune hepatitis	Not yet recruiting	Nanjing Medical University, China
**NCT03185000**	I/II	Autologous polyclonal Tregs	Peripheral blood	0.5–1, 3–5 and 8–10 × 10^6^ cells/kg	NA	Crohn’s Disease	Not yet recruiting	King’s College London
**NCT02772679**	I	Autologous polyclonal Tregs + IL-2	NA	Cohort 1: 3x10^6^ cells Cohort 2: 20x10^6^ cells	NA	T1DM	Active, not recruiting	California, United States
**NCT02932826**	I/II	UCB-Treg	Umbilical cord blood	1–5x10^6^/kg	Insulin	T1DM	Recruiting	Hunan, China

UCB-Treg, Regulatory T cells expanded from umbilical cord blood; Type 1 Diabetes Mellitus, T1DM; NA, Not available.

In another study (first-in-man treatment of T1D by Tregs), published in 2014, after a one year follow-up of 12 T1D patients infused with autologous Tregs, it was shown that eight out of 12 patients had low insulin requirement and high C-peptide level (which reflects *β*-cell mass) and two patients became completely independent from insulin in 1 year. On the contrary, non-treated controls had insulin requirements and lower C-peptide levels compared with treated subjects. Therefore, Treg therapy with CD4^+^CD25^high^ CD127^−^ Tregs resulted in the increased survival of pancreatic islets in T1D patients (174). Nonetheless, the therapeutic effect of Tregs would decrease over time. Even though, the majority of patients have been in remission throughout one year follow-up, a steady T1D development and Treg number reduction (return to the baseline values) was reported with time. These data indicate that *ex vivo* expanded Tregs are short-lived following administration as they passed multiple cycles of divisions *in vitro*. In addition, peripheral tissues homing may account for the reduction in Treg numbers in treated patients. The collapse in Treg counts may be attributed to Treg exhaustion in a long term because of prolonged activation during autoimmune response suppression. Furthermore, Treg stability is a current debate.

The application of Treg-promoting in conjunction with Treg therapy may enhance the suppressive function as well as Treg number with ameliorated patient outcomes namely rapamycin, IL-2, *etc*. As an example, the administration of IL-2 in combination with polyclonal Tregs can be designed for phase I clinical trial (NCT02772679). As outlined previously, low-dose IL-2 therapy exclusively can cause expansion of Tregs *in vivo*. The number of Treg and function is predicted to be increased by concurrent administration of polyclonal Tregs and low-dose IL-2 (152). One ongoing study (NCT02704338), is assessing the safety and efficacy of Tregs in treating autoimmune hepatitis. In this clinical trial, CD4^+^CD25^+^CD127^−^ Tregs separated from peripheral blood samples of autoimmune hepatitis patients and expanded by IL-2 and CD3/28 beads. These cells were then administered to patients with single infusion. The number of Tregs in patients monitored at different periods and their suppressive mechanisms were studied. In order to determine the efficacy of this therapy, both the function and biopsy of the liver will be evaluated.

In fact, Treg administration in combination with other therapies can be an effective strategy to treat autoimmune diseases, and further investigations in achieving the desired outcome is required.

To date, numerous polyclonal Treg-based clinical trials have been performed in patients with different autoimmune diseases that exhibit promising effects on modulating immune responses. Further investigations are required and underway as the clinical trials NCT02428309, NCT03011021 and, NCT03239470 can verify the therapeutic efficacy of Tregs in autoimmunity (6) (**Table 3**).

The application of a high number of polyclonal Tregs without any selection for Ag specificity in these clinical trials, enhance the risk of systemic immunosuppression (175) and make the patients more prone to infections and tumors. Given the plasticity and instability of pTreg in the inflamed tissues, this method becomes more challenging. The reprogramming of pTregs to pathogenic effector T cell can occur in chronic inflammation (176). At present, there are two approaches that are used in achieving antigen-specific Treg including engineered transduced TCRs and CARs (177).

#### Antigen-Specific Treg Therapy

Treg therapy can be improved through cellular engineering to be autoantigen-specific by which their potency and suppressive effect would be promoted (178).

In the Crohn’s And Treg Cells (CATS1) study, Desreumaux et al. advanced the notion of adoptive transferring of antigen-specific Tregs in the treatment of autoimmune disease (179). CATS1 study is the first clinical trial of adoptively transferred, ovalbumin (OVA)-specific Tregs performed in patients with Crohn’s disease. In this study, patients received a single dose of 10^6^, 10^7^, 10^8^, or 10^9^ autologous ova-Tregs. Treg dose-related efficacy and tolerable safety profile in patients with Crohn’s disease have demonstrated the significant improvement in survival rate (40%) of these patients (179). The variation in responses was evident among dose groups. The most significant effect was observed in the group who received 10^6^ autologous ova-Tregs.

Another way to treat specific autoimmune disease is utilizing cell-based therapy in which Tregs through retroviral or lentiviral transduction express an autoantigen-specific TCR (152).

The use of genetically engineered NOD (non-obese diabetic) mouse model to express the diabetogenic TCR represents that comparatively few antigen-specific Tregs, but not of polyclonal Tregs, are adequate to halt and even reverse T1D (180).

Compared to polyclonal Tregs, very few antigen-specific Tregs are required to ameliorate autoimmune disease. However, it is still a matter of debate to identify a proper, high-affinity, autoantigen-specific TCR which can transduce into a Treg for several autoimmune diseases with ill-defined dominant epitopes (152). In addition, as antigen-specific Tregs are mostly presented in tissues, isolating them and identifying their cognate antigens would be strenuous (178).

However, the limitations of the present methods are isolating sufficient number of autologous antigen-specific Tregs and expand them; novel techniques which are based on producing significant amount of antigen-specific Tregs are being investigated. These strategies, which may push the boundaries, involve transferring TCR genes into expanded polyclonal-Tregs *via* lentiviral transduction, gene-editing of Foxp3 in antigen-specific CD4^+^ T cells, and reprogramming of effector T-cells to Treg-like cells by CRISPR/Cas9-mediated integration of a Foxp3-transgene (181–183).

The human Tregs transduced with a factor VIII (FVIII)-specific TCR are able to suppress FVIII-specific conventional T cells as well as anti-FVIII antibody secretion from primed splenocytes (184).

By the same token, it is demonstrated that regulative potency of Tregs transduced with islet-specific TCR was stronger compared with that of polyclonal Tregs *in vitro* (185).

#### CAR-Treg Therapy

It has been demonstrated that CAR-Tregs have increased potency in various experimental models of autoimmune diseases, notably colitis and experimental autoimmune encephalomyelitis (186, 187).

The CAR-Treg application was initially conducted in an animal model in 2008. In this approach, 2,4,6-trinitrophenol CAR-Treg suppressed the severity of 2.4.6-trinitrobenzene sulfonic acid-induced colitis, whereas such effect was not observed in polyclonal Treg (188). Moreover, the study demonstrated that genetically modified Tregs may expand in an antigen-specific manner and preferentially home to inflamed colonic mucosa. Another study has revealed that Treg expressing a CAR specific for carcinoembryonic antigen (CEA) could improve ulcerative colitis and suppress the colorectal cancer progression (189). In a study, the engineered myelin oligodendrocyte glycoprotein (MOG)-specific CAR Tregs were developed to inhibit EAE as a model of MS in humans. The genetically modified Tregs manifested suppressive potential *in vitro*, impacting on diminishing disease symptoms, and the capacity to accumulate in different areas of the brain followed by the intranasal administration in mice with active EAE (186). The success of the study inspired some investigations to assess the potential of CAR Tregs in treating some types of other autoimmune diseases and the outcome was significant (190).

In spite of the promising results, there are some hurdles in employing CAR-Tregs: It is intricate to choose the optimal target for CAR-Tregs and also this approach is associated with some remarkable limitations including the time-consuming process and off-target effects (126). The autoantigen should be expressed uniquely at the site of autoimmunity. Otherwise, the antigen-specific response would not be effective resulting in systemic hyper-activation of the CAR-Tregs and causing side effects such as general immunosuppression. Additionally, Treg accumulation in healthy tissues may create a context for occurrence and development of cancer and pathogen persistence, yet presumably this issue has not been resolved by experimentation so far (191).

Using CAR-Tregs is superior to TCR-transgenic Tregs, as CAR-Tregs is non-HLA-limited and less IL-2-dependent. It is not obvious whether high affinity and downstream signaling of CARs would be perfect for function of Treg (178). The cell lineage and phenotypic stability of the therapeutic cells are considered a safety problem in cell-based therapy. For a successful therapy, Tregs need to maintain their specificity, stability, suppressive capacity, and also their persistency over the long term (152).

Eventually, CAR-Treg exhaustion is another disadvantage which may limit the development of CAR-Tregs (192). To address these difficulties, the incorporation of CD28 or CD137 costimulatory domains with second-generation CARs enhance the effect of CAR Tregs. To choose the ideal costimulatory signals for optimal CAR-Treg suppression, further research is needed (126).

## Role in Cancer

The involvement of Tregs in tumor immunity has been established since 1991 (193). Studies indicate that the growth of syngeneic tumors (such as leukemias, myeloma and sarcomas) in mice is prevented by administration of anti-CD25 mAb (194, 195).

Clinical studies have shown that the determined number of pTregs in the blood and tumor tissues of cancer patients with squamous cell carcinoma is more than in healthy individuals (196). Moreover, inhibitory surface markers such as CD39, CD73, LAP, GARP, and COX-2 have a higher membrane expression on the pTreg of cancer patients (197). In addition, the function and phenotype of tumor residing Tregs are modified compared to circulating Tregs (197) In other words, the expression of some inhibitory receptors such as CTLA-4, TIM-3, PD-1, and CD39 increases on the surface of intratumoral Tregs (198, 199). To sum up, these clinical results support the notion that sites of tumor have activated Tregs, and these cells have a significant immunosuppressive potential (193) and making cancer therapy more challenging.

Also, in a study conducted by, Jie et al., it was demonstrated that in head and neck squamous cell carcinoma (HNSCC) patients, intratumoral Tregs are more immunosuppressive than peripheral blood Tregs. Additionally, it was indicated that most intratumoral Tregs, co-express CTLA-4 and CD39 (198). As a result, the blockade of CTLA-4 or inhibiting CD39 activity could potentially help in the treatment of these types of cancer.

Adenosine (a product of ATP degradation by CD39 and CD73) has an important role in suppressing effector T cells and has an important effect in the tumor environment. In the tumor niche, adenosine results in the increased migration of effector T cells to the tumor environment and subsequently suppressing them. Adenosine, also enhances the differentiation (from CD4^+^ effector T cells), the proliferation of Tregs and inhibitory mechanism of Tregs and myeloid-derived suppressor cells (MDSCs) (197, 200). The other important effect of adenosine is the augmentation of metastasis *via* increasing pro-metastatic and proangiogenic factors. In other words, adenosine has direct effects on vascular endothelial cell proliferation or indirect effects on vasculature *via* polarization of tumor cells or immune cells within the tumor environment (201, 202). It is demonstrated that PGE2 levels are high in the tumor environment (203). PGE2, has an immune-suppressive effect on effector T cells and can induce Tregs (204) and promote the secretion of IL-10 and TGF-*β* from Tregs (205).

PGE2, is an important product of cyclooxygenase 2 (COX-2) (206). COX-2 shows an increased expression in various tumors (207–211) which is also linked with poor prognosis (211). It is demonstrated that the co-culture of Tr1 cells (a peripherally derived Treg) with COX-2^+^ tumors, can induce COX-2 expression in these cells and can also induce PGE2 and adenosine secretion (207). Both, adenosine and PGE2 immunosuppressive effects, are mediated by G protein-coupled receptors, namely A2AR, A2BR (adenosine receptors) and EP1-4 (PGE2 receptor). They have the same inhibition mechanism for effector T cells in the tumor environment. Meaning, both factors can increase cytosolic cAMP levels and PKA type I activation, resulting in the suppression of responder T cells, down-regulating anti-tumor responses, and tumor progression (197, 212).

Furthermore, it is shown that the number and activity level of circulating Tregs expressing CD4^+^CD39^+^ and/or CD4^+^COX-2^+^ increase in patients with advanced disease. In other words, PGE2 and adenosine pathways cooperate to mediate the maximum immunosuppressive effects of Tregs in the tumor environment (213).

Other suppressive mechanisms in regard to Tregs’ roles are: the secretion of suppressive cytokines such as IL-10, TGF-*β*, killing activated CD8^+^, utilizing Neuropilin/semaphorin-4a pathway (197), the consumption of available IL-2 and releasing GrB, exosome production and delivery of the pTreg products to effector T cells, diminishing anti-tumor immunity (86, 197).

The involvement of Treg components in tumor progression suggests that the frequency of Tregs in tumors or in the periphery area could be used for prognosis or potential cancer biomarkers. However, because of Tregs heterogeneity and lack of unique markers for its isolation, its application as a prognostic cancer marker is not easy (197).

### Treg as an Emerging Therapeutic Target for Cancer Therapy

**Figure 6:**
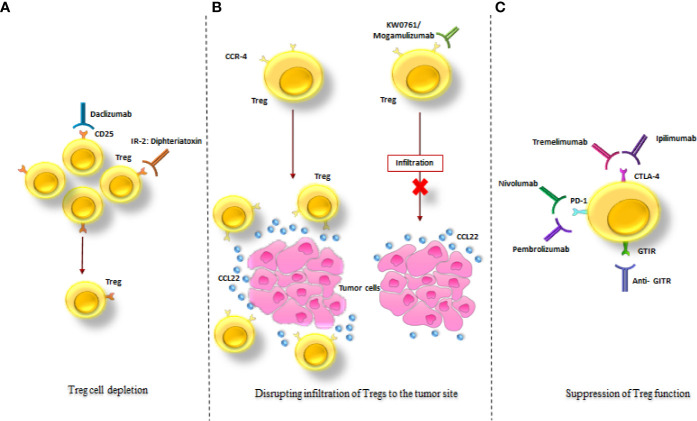
Treg-targeted therapeutic strategies in cancer treatment. The schematic drawing of the three major mechanisms in tumor treatment *via* targeting Tregs; **(A)** Treg depletion *via* anti-CD25 monoclonal antibody (Daclizumab) and an IL-2: diphtheria toxin fusion protein (Denileukin Diftitox). **(B)** Disruption of Tregs infiltration to the tumor site *via* KW0761/Mogamulizumab. **(C)** Suppression of Treg function *via* Ipilimumab, Tremelimumab, Pembrolizumab, Nivolumab and Anti- GITR. All of these strategies resulted in diminution of the immunosuppressive effects of Tregs in the tumor site (as mentioned in the text).

There are few methods for Treg modulation with the aim of clinical cancer therapy:

1. Treg depletion
2. Disrupting infiltration of Tregs to the tumor site
3. Suppression of Treg functions (8, 9) (**Figure 6**, **Supplementary Table 2**).

#### Tregs Depletion

Treg depletion strategies are performed by targeting CD25 (one of the main surface marker of Tregs) by an anti-CD25 mAb (Daclizumab) and an IL-2-diphtheria toxin fusion protein (Denileukin Diftitox) (9) (**Supplementary Table 2**). In 2012, Sampson et al. demonstrated that Daclizumab given concomitantly with epidermal growth factor receptor variant III (EGFRvIII) targeted peptide vaccination is correlated with increased humoral immunity in patients with glioblastoma (214). This suggests that there is a reverse relationship between Treg frequency and vaccine-stimulated antibody levels.

In another study, it was shown that the administration of a single dose of Daclizumab to metastatic breast cancer patients resulted in Treg depletion in peripheral blood and effective generation of cytotoxic T lymphocytes after a cancer antigen peptide vaccine administration (215).

However, in another study in metastatic melanoma patients, the combinational therapy of Daclizumab with DC vaccination (pulsed with tumor peptide) did not augment the efficacy of the DC vaccine (216). Hence, the depletion of CD4^+^Foxp3^+^CD25^high^ Tregs from the peripheral circulation of the patients didn’t increase the efficacy of the DC vaccination against the tumor (216).

Notably, another study indicated that despite the beneficial effects of Denileukin Diftitox in the treatment of T cell lymphoma and renal cell carcinoma patients (217); this therapy was not successful in the treatment of melanoma patients and could not eliminate Tregs (218).

In addition to the aforementioned, a phase II trial study in advanced melanoma patients demonstrated that the single dose of denileukin diftitox did not deplete Tregs. Also, vaccine-induced T cell responses did not increase. Hence, no clinical improvements were determined (219).

Denileukin Diftitox is a fusion protein consisting of IL-2 and diphtheria toxin which reduces CD25^+^ cells (220). Accordingly, denileukin diftitox cannot differentiate between Tregs and activated encoding IL-2Rs; therefore concurrent depletion of both effector T cells and Tregs occurred (214).

It should be taken into account that CD25 expression also induces on activated effector T cells; therefore both concurrent depletion of effector T cells and Tregs occurred (221).

RG6292 is the first anti-human CD25 antibody created to preferentially reduce Tregs but entirely maintaining IL-2 signaling and CTL activity. In pre-clinical tests, a single-dose of the RG6292 efficiently enhanced the elimination of established tumors in some tumor mouse models. It is anticipated that RG6292 release the capacity of selective depletion of Treg whereas permitting limitless access of IL-2 to CTLs; consequently it is clinically advantageous to other Treg depleting antibodies (222). The safety and tolerability of RG6292 are being assessed in clinical trials in patients with progressive solid tumors (NCT04158583).

Also, it is important to mention that as well as Treg specificity, dosage and timing are considerably significant for the immunotherapeutic advantage of transient Treg depletion (223).

#### Disrupting Infiltration of Tregs to the Tumor Site

Studies regarding Treg chemotaxis through chemokine ligand 1 (CCL1)‐chemokine receptor 8 (CCR8) and CCL22‐CCR4 into the tumor microenvironment (TME) have been performed. Inhibiting the interaction of chemokine and chemokine receptor mitigates Treg aggregation in the TME, resulting in enhancing antitumor immune responses (41). This suggests that targeting these pathways could be potentially effective for cancer therapy (224) (**Supplementary Table 2**). Recently, a study on lung and esophageal cancer patients, conducted with KW-0761/mogamulizumab infusion (a humanized anti-human CCR4 mAb that has antibody-dependent cellular cytotoxicity activity) and its effects investigated on these patients (225). Tolerability and safety of the mAb infusion are demonstrated in a dose range between 0.1 mg/kg and 1.0 mg/kg without any dose-limiting toxicity. In this phase Ia study, throughout the treatment only four in 10 patients exhibited stable disease and were the long-term survivors. Monitoring Foxp3^+^ Tregs showed effective depletion of these cells (even in low dose) with a limited reduction of Th1 CD4^+^ T cells and CD8^+^ T cells and a significant reduction of Th2 and Th17 CD4^+^ T cells. The study indicated that the depletion of Treg might give rise to increase of immune responses followed by KW-0761 infusion, yet no clinical responses were discovered in patients (225).

CCR4 belongs to the seven transmembrane G-protein-coupled receptor which is expressed by different cells including Th2, Treg, memory T cells, among which Th2 and Treg are preferentially and considerably expressing CCR4 (226). Anti-CCR4 mAb therapy can result in off-target which may cause off-target effects, and Treg depletion may cause impaired immune responses to infection (227). Currently, additional clinical trials along with an immune checkpoint inhibitor are being studied (228, 229).

Recently, chemokine receptor named CCR8 (a receptor for CCL1) has been discovered which is expressed on intratumoral Tregs in several cancers, notably breast, colon, lung, and renal cell carcinoma (230–232) with low expression in Th2 and monocytes with small proportions of expression in Th2 and monocytes (233). As well, it has been shown that in muscle-invasive bladder cancer, CCR8 was particularly expressed by Foxp3^hi^ Tregs but not by Foxp3^mid^ and Foxp3^neg^ effector T cells. Expression in the peripheral blood, thymus, and skin resident T cells was lower. The same pattern was seen in mouse tumor model (231). The high expression of CCR8 in tumor-infiltrating Tregs demonstrates it may be a potential therapeutic target to suppress Tregs homing to tumor sites independent of other effector cells that do not express CCR8 (234, 235). CCR8^+^ Tregs are a stable subtype of Tregs with enhanced immunosuppressive properties (230), and in tumor sites where CCR8^+^ Tregs are plentiful imply the inferior prognosis (231, 233). A recent *ex vivo* experiment has demonstrated that blockade of CCR8 can destabilize Treg, reactivates the antitumor immunity, and strengthen the efficacy of anti-PD-1 therapy (230).

BMS-986340 is a newly developed anti-CCR8 mAb which reduces sizeable CCR8^+^ Treg in a human tumor explant. Depletion of CCR8^+^ Treg exerts vigorous antitumor effect independently or in combination with PD-1inhibitor (231).

These outcomes favor additional clinical assessment of CCR8 reduction along with immune checkpoint inhibitors as a new cancer immunotherapy.

#### Suppression of Treg Function

Targeting inhibitory receptors/checkpoint molecules like CTLA-4 and PD-1 has promising effects in cancer therapy. Moreover, the FDA approved human mAbs that target CTLA-4 are Ipilimumab and Tremelimumab (236), and those that target PD-1 are Nivolumab and Pembrolizumab (236).

Whereas CTLA-4 is constitutively expressed in Tregs; this is only upregulated on activated conventional T cells at lower level compared to Tregs even in the tumor (85). As a result of the high CTLA-4 expression on Tregs and its significant role in the suppressive mechanisms of Tregs, it appears that the therapeutic targeting of this receptor could improve cancer therapy (237) (**Supplementary Table 2**).

Ipilimumab, is an IgG1 isotype which is FDA approved for the treatment of metastatic melanoma and it is under clinical investigation in different phases of various tumors like renal cell cancer, prostate, and lung cancers (238).

The X-ray crystal structure of the Ipilimumab in complex with CTLA-4 indicates that Ipilimumab binding an epitope overlaps the residues form B7 binding site, and the direct steric overlapping between Ipilimumab and the B7 ligands plays a principal mechanistic role in Ipilimumab function (239). CTLA-4 blockade stimulates anti-tumor immunity by increasing the expansion of CD45RO^+^ICOS^+^PD-1^low^TBET^+^ Th1-like CD4^+^ effector and also exhausted-like CD8^+^ T cells (240). Interestingly, *ex vivo* studies have shown that the presence of human IgG1 isotype in Ipilimumab resulted in ADCC-mediated lysis of Tregs by Fc*γ*RIIIA (CD16) expressing non-classical monocytes in monocyte/T-cell cocultures (241). Rosskopf et al. showed that the inhibitory effects of Ipilimumab were eliminated by reducing CD16^+^ cells (242). Although, it is still not apparent whether the antitumor effect of Ipilimumab in humans is correlated with the CTLA-4 molecule blockade or reduction in CTLA-4^+^ cells by ADCC (85, 243).

In addition to the aforementioned, Yang et al. showed that Ipilimumab resulted in metastatic renal cell cancer regression (238). However, this regression is exceedingly connected with immune-related adverse events (irAEs).

In another study it was shown that CTLA-4 blockade leads to the enhanced proportion of IFN-*γ* producing CD4^+^ ICOS^hi^ effector T cells to Tregs in peripheral blood and tumor tissues of patients with bladder cancer (244). It has also been revealed that by Ipilimumab therapy, Foxp3^+^ Tregs in the tumor tissues of melanoma patients remarkably decreased specifically in clinical responders (241). Additionally, an increased ADCC activity in melanoma patients who carry a high affinity genetic variant of Fc*γ*RIIIA (CD16) has improved remarkably their chances of survival and clinical responses to Ipilimumab compared to patients with a low affinity variant (243). A new study revealed that Fc-engineered anti-CTLA-4 mAb (with high ADCC activity) was able to increase the anti-tumor immunity *in vitro* in humans and *in vivo* in mice by decreasing CTLA-4^hi^ effector Tregs, whereas anti-CTLA-4 mAbs with much less or no ADCC activity did not exhibit the increment (85). The therapeutic outcomes of patients with highly immunogenic tumors have been improved through ADCC enhancement either by Fc optimization or the existence of Fc*γ*R variants with high binding affinity (243). The mutational burden along with Fc*γ*R polymorphism status should be taken into account in choosing patients who might react to anti-CTLA-4.

It was reported in a study that following robust upregulation of CTLA-4, stimulated CD4^+^ effector T could be potentially the target of Ipilimumab-mediated ADCC. Ipilimumab gave rise to considerable decrease in proliferation of CD4^+^ T cells and secretion of cytokine. More studies are required to determine if Ipilimumab may reduce CTLA-4-expressing CD4^+^ effector T cells *in vivo* (242). CTLA-4 is expressed less on CD8^+^ than on CD4^+^ T cells, and in different experiments the failure of CD8^+^ T-cell response by Ipilimumab has not been revealed (245–247).

Other anti-CTLA4 mAbs, Tremelimumab has a human IgG2 isotype and has no ADCC capacity (248). This drug is currently under clinical trial investigation for melanoma (249) and malignant mesothelioma (250).

In an ongoing phase II clinical trial in Hepatocellular carcinoma (NCT02519348) and phase III in head and neck cancer (NCT02369874), the combined effects of Tremelimumab and MEDI4736 (anti-PD-L1 antibody Durvalumab) are investigated against MEDI4736 or Tremelimumab monotherapy. Also, in another study working on the safety and anti-tumor activity of Durvalumab and Tremelimumab, it was shown that 20 mg/kg of Durvalumab every 4 weeks plus Tremelimumab (1 mg/kg) had a manageable tolerability profile in non-small cell lung cancer (251).

It has been found that combining anti-CTLA-4 antibodies with immunotherapy, chemotherapy or radiotherapy has a great influence to ameliorate long-term survival rates of patients with various types of tumor malignancies (252).

In comparison with anti-PD-1 treatment, CTLA-4 targeting is associated with two challenges of suboptimal efficacy and elevated toxicity (253). Increased activity can be seen in Ipilimumab therapy combining with other immunomodulators/checkpoint antibodies including those targeting PD-1 or Tremelimumab and anti-PD-L1 (239). PD-1 is expressed by activated Tregs as well as effector T cells (254). The impact of PD-1 blockade on Tregs in which comparable expression levels of PD-1 and effector T cells are equivalently expressed, is still not obvious. As Tregs demonstrate these equivalent expression, especially in the TME, and their function and survival are contingent on TCR and CD28, PD-1 blockade may stimulate the immune inhibitory function of Tregs (41).

The mechanisms underlying responses to anti-PD-1 immunotherapy solely in some patients have been mysterious (255). A recent study indicates that the balance of PD-1 expression between CD8^+^ T cells and intratumoral Tregs can be a predictive biomarker for the efficacy of PD-1 blockade therapy (256). As PD-1 is largely expressed in intratumoral CD8^+^ T cells, anti-PD-1 immunotherapy converts PD-1^+^CD8^+^ T cells to CD8^+^ effector T cells, giving rise to tumor regression. In contrast, if PD-1 is largely expressed by intratumoral Treg, anti-PD-1 immunotherapy converts them into activated Tregs, resulting in tumor progression (255). Obviously, further research is needed to ascertain the requirements of PD-1-mediated signaling to selectively induce Treg generation or inhibit their activation and also increase their suppression potential. Besides targeting the inhibitory receptors of Tregs, costimulatory molecules of Tregs like GITR (glucocorticoid-induced TNF receptor family-related protein) and OX40 can be also used for tumor therapy (**Supplementary Table 2**). Comparable to anti-CTLA-4 mAb, it has been proved that mAbs against OX-40 and GITR are contingent on ADCC-mediated reduction of Tregs to practically inhibit the growth of tumor (257).

GITR is permanently expressed on Tregs and has inducible expression on conventional T cells (258, 259). The treatment of advanced tumors with agonistic anti-GITR mAb in mouse models indicated not only increased the level of IFN-*γ* producing CD8^+^ and CD4^+^ effector T cells infiltration, but also attenuated Treg mediated suppression (260).

INCAGN01876 is an anti-GITR agonistic (IgG1) mAb developed with the aim of advancing malignancy treatment. INCAGN01876 without cross-reaction with other TNFR family members binds strongly to human and non-human primate GITR (261).

Apart from that, another anti-GITR mAb, TRX518, was the first anti-GITR mAb introduced to the clinic in 2010, for malignant melanoma and recently in solid tumors (**Supplementary Table 2**).

In summary, pharmacological activation of GITR has some effects on Tregs, such as: selective depletion of intratumoral Tregs by ADCC mechanism, attenuation of immunosuppressive activity of Tregs and, enhancing anti-tumor immunity by shifting the Teff/Tregs ratio (258, 262).

It has been revealed anti-GITR mAbs associated with a manageable safety profile. However, it appeared not to be effective as a monotherapy. Also, there are other anti-GITR mAbs including MK-4166, MK-1248m, MEDI1873, *etc*. alone or particularly in combination with other drugs under clinical investigations (258, 259).

OX40 has a high expression level on resting and activated Tregs. However, its expression is transiently induced on effector T cells (224). Some anti-OX40 compounds affect T cells by decreasing Tregs’ suppressive function on T cells and increasing the survival of various effector T cell subsets (263). There are different mAbs targeting OX40; one of them is OX40L-Fc, and the others are agonistic anti-OX40 antibodies (263).

The safety is the most important result from clinical trials using OX40-targeted drugs once employed both as monotherapy or combined with other immune-checkpoint inhibitors. OX40-targeted therapy demonstrated profound outcomes in tumor-bearing mice; however, clinical data in human showed the effect of monotherapy was moderate (257).

Administration of these Abs, have the potential for evoking anti-tumor immune response (8) and their influences on regression of diverse types of tumors is under investigation.

Although the successful targeting of immune checkpoint has recently been shown great merit in cancer therapy, adverse side effects of this drugs (fatigue, fever, diarrhea, skin rash, itching and nerve inflammation, *etc*.) have been frequently reported in patients caused by activation of non-tumor-specific T cells by the immune blocking antibodies.

As a consequence, intense researches are underway to recognize promising biomarker candidates for immune checkpoint targeting therapy that will boost specificity, efficacy and selectivity of the treatment, and diminish side effects of these drugs (252).

Considering the current knowledge, there are ongoing experiments to discover a highly specific Treg marker. Another approach to boost tumor immunity could be through combination therapy focused on both Tregs and effector T cell by changing numerical and functional equilibrium between the two populations simply by reducing Tregs or mitigating inhibitory function of Tregs and concurrently increase effector T cell proliferation or enhance their effector activity (8).

## Conclusion

Since the discovery of Tregs as a key component of immune homeostasis that maintains self-tolerance, immunological applications of Tregs in opposite clinical contexts, autoimmunity, transplantation and antitumor immunity, have been explored extensively.

Manipulation of Tregs is under intense scientific and commercial scrutiny as a novel therapeutic strategy for treatment of different diseases.

Clearly, many questions remain regarding optimization of strategies that target Tregs. Further basic and translational research is warranted, and an extensive understanding of Treg development, maintenance and function, could potentially lead to increases in the efficacy of Treg-targeted therapies, development of successful therapeutic interventions in these disorders and reduce the risk of adverse effects of such treatments.

## Author Contributions

FB, MM, and ES-P participated in manuscript writing, editing, and figure development. FB, MV and ES-P came up with the design and conceptualization of the review. SJ, MV, and AF participated in manuscript writing. All authors contributed to the article and approved the submitted version.

## Funding

The corresponding author acknowledges the financial support from King’s College London, Medical Research Council Centre of Transplantation, the British Heart Foundation, the Centre of Excellence in Medical Engineering funded by the Wellcome Trust and the EPSRC under grant number WT088641/Z/09/Z, the King’s College London and University College London Comprehensive Cancer Imaging Centre funded by CRUK and EPSRC in association with the MRC and DoH (England), and the National Institute for Health Research (NIHR) Biomedical Research Centre based at Guy’s and St Thomas’ NHS Foundation Trust and King’s College London. The views expressed are those of the authors and not necessarily those of the NHS, the NIHR or the Department of Health.

## Conflict of Interest

FB and SJ were employed at Aryogen Pharmed.

The remaining authors declare that the research was conducted in the absence of any commercial or financial relationships that could be construed as a potential conflict of interest.
