# Supplementary material for: The Therapeutic Potential of Regulatory T Cells: Challenges and Opportunities
Source: Front Immunol. 2021 Jan 15;11:585819. doi: 10.3389/fimmu.2020.585819 (PMC7844143; doi:10.3389/fimmu.2020.585819)
Supplement: Supplementary file 1 [file Table_1.docx]

**Supplementary Data**

| Supplementary Table 1. Ongoing Treg-based clinical trials in Transplantation | | | | | | | | | |
| --- | --- | --- | --- | --- | --- | --- | --- | --- | --- |
| Study ID | Phase | Intervention | | Source | Dose | Drugs | Condition | Status | Location |
| NCT03284242 | NA | Autologous polyclonal Tregs | Blood cells | | NA | Tacrolimus and Mycophenolate acid with or without Steroids | Kidney Transplant | Recruiting | Kentucky, United States |
| NCT01624077 | I | Autologous allospecific Tregs | Peripheral blood | | 1 x 10^6^ cells/kg | Tacrolimus | Liver Transplant | Unknown | Nanjing, Jiangsu, China |
| NCT02091232 | I | Belatacept-conditioned Tregs (from donor & recipient) | Peripheral blood | | NA | NA | Kidney Transplant | Active, not recruiting | Massachusetts, United States |
| NCT03943238 | I | Recipient Treg and Donor HSCs | NA | | escalated dose of recipient  Tregs starting at 25 x 10^6^/Kg | TLI, ATG | Kidney Transplant | Not yet recruiting | United States |
| NCT02188719 | I | Donor-alloantigen-reactive Treg | NA | | 50, 200, 800 x 10^6^ cells | ATG, Everolimus,  Tacrolimus, Mycophenolate mofetil, Prednisone, Acetaminophen,  Diphenhydramine,  Anti-Infective Prophylaxis | Liver Transplant | Terminated | United States |
| NCT03654040 | I/II | Donor alloantigen-specific Tregs | NA | | 100-500 x 10^6^ cells | Cyclophosphamide, mesna, Everolimus | Liver Transplant | Withdrawn | California, United States |
| NCT03577431 | I/II | Donor alloantigen-specific Tregs | NA | | 2.5-500 x 10^6^ cells | Cyclophosphamide, mesna, everolimus | Liver Transplant | Recruiting | Massachusetts, United States |
| NCT03867617 | I/II | Recipient Tregs + Donor Bone Marrow Infusion | NA | | NA | Tocilizumab | Kidney Transplant | Recruiting | Vienna, Austria |
| NCT02711826 | I/II | Autologous polyclonal   Tregs | NA | | 550 ± 450 x 10^6^ cells | Everolimus,  Tacrolimus,  Mycophenolate mofetil, Acetaminophen,  Diphenhydramine | Kidney Transplant | Recruiting | United States |
| NCT01446484 | I/II | Autologous  Tregs | Peripheral blood | | 200 x 10^6^ cells | AlemtuzumabMycophenolate mofetil, Sirolimus, Tacrolimus Cyclosporine, Everolimus | Kidney Transplant | Unknown | Moscow, Russian Federation |
| NCT03444064 | I | Autologous polyclonal  Treg | NA | | 400-1600 x 10^6^ cells | Tacrolimus and Sirolimus | Islet Transplantation | Recruiting | Alberta, Canada |
| TLI, Total lymphoid irradiation; ATG, Anti-thymocyte globulin | | | | | | | | | |

| Supplementary Table 2. The last clinical trial status of mAbs targeting Tregs in cancer | | | | | | | |
| --- | --- | --- | --- | --- | --- | --- | --- |
| Target | **Agent** | **Main**  **mechanism** | **ID** | **Tumor types** | **Phase** | **Development**  **stage** | **Location** |
| CD25 | Daclizumab | Treg depletion | NCT01418430 | Leukemia/Lymphoma | IV | Completed | King's College, London |
|  |  |  | NCT00847106 | Melanoma | II | Completed | Radboud University |
|  | Denileukin Diftitox |  | NCT00050999 | T cell lymphoma | IV | Completed | Eisai Inc.  US |
|  |  |  | NCT00726037 | Metastatic pancreatic cancer | II | Terminated | Loyola University, US |
|  |  |  | NCT00880360 | Ovarian cancer |  | Completed | Texas, US |
|  |  |  | NCT00082914 | Metastatic melanoma or metastatic kidney cancer |  | Completed | National Cancer Institute (NCI), US |
|  |  |  | NCT00425672 | Breast cancer |  | Completed | University of Washington |
| CCR4 | KW-0761/ mogamulizumab | Disrupting infiltration of Tregs to the tumor site | NCT01728805 | Lymphoma | III | Active, not recruiting | Kyowa Kirin Pharmaceutical Development, Inc. |
|  |  |  | NCT02946671 | Gastric Cancer  Esophageal Cancer  Lung Cancer  Renal Cancer  Oral Cancer | I | Completed | Osaka University, Japan |
| CTLA-4 | Ipilimumab | Suppression of Treg function | NCT01856023 | Melanoma | IV | Terminated | Prometheus Laboratories |
|  |  |  | NCT02068196 | Melanoma |  | Active, not recruiting | Oslo University Hospital |
|  |  |  | NCT02982954 | Renal Cell Carcinoma |  | Active, not recruiting | Bristol-Myers Squibb |
|  |  |  | NCT02869789 | Lung Cancer |  | Recruiting | Bristol-Myers Squibb |
|  | Tremelimumab |  | NCT00257205 | Melanoma | III | Completed | AstraZeneca |
|  |  |  | NCT03288532 | Renal Cell Carcinoma |  | Recruiting | University College, London |
|  |  |  | NCT02516241 | Urothelial Cancer |  | Active, not recruiting | AstraZeneca |
|  |  |  | NCT02551159 | Squamous Cell Carcinoma of the Head and Neck |  | Active, not recruiting | AstraZeneca |
|  |  |  | NCT02352948 | Non-small cell lung cancer |  | Active, not recruiting | AstraZeneca |
|  |  |  | NCT03298451 | Hepatocellular carcinoma |  | Active, not recruiting | AstraZeneca |
| PD-1 | Nivolumab |  | NCT02626065 | Melanoma | IV | Unknown | Hospices Civils de Lyon |
|  |  |  | NCT02869789 | Lung cancer |  | Recruiting | Bristol-Myers Squibb |
|  |  |  | NCT02596035 | Renal cell carcinoma |  | Active, not recruiting | Bristol-Myers Squibb |
|  |  |  | NCT03444766 | Non-small cell lung cancer  Kidney cancer |  | Active, not recruiting | Bristol-Myers Squibb |
|  | Pembrolizumab |  | NCT03715205 | Metastatic melanoma or Non-small cell lung cancer | IV | Recruiting | Merck Sharp & Dohme Corp. |
|  |  |  | NCT03891979 | Pancreatic adenocarcinoma |  | Suspended | NYU Langone Health |
|  |  |  | NCT03134456 | Non-Small Cell Lung Carcinoma |  | Unknown | Samsung Medical Center |
| GITR | INCAGN01876 |  | NCT02697591 | Metastatic cancer | I/II | Active, not recruiting | Incyte Biosciences International Sàrl |
|  |  |  | NCT03277352 | Metastatic cancer | I/II | Active, not recruiting | Incyte Biosciences International Sàrl |
|  |  |  | NCT04225039 | Glioblastoma | II | Not yet recruiting | University of Pennsylvania |
|  |  |  | NCT03126110 | Metastatic cancer | I/II | Recruiting | Incyte Biosciences International Sàrl |
|  | TRX518 |  | NCT01239134 | Malignant melanoma or other solid tumors | I | Completed | New York, US |
|  |  |  | NCT03861403 | Breast Cancer | I/II | Active, not recruiting | US |
|  |  |  | NCT02628574 | Solid Tumors | I | Active, not recruiting | US |
| OX40 | Agonistic anti-OX40 antibodies |  | NCT01689870 | Melanoma | II | Withdrawn | Ludwig Institute for Cancer Research |
|  |  |  | NCT03390296 | Leukemia |  | Recruiting | M.D. Anderson Cancer Center |
|  |  |  | NCT03217747 | Prostate Cancer |  | Recruiting | M.D. Anderson Cancer Center |
|  |  |  | NCT03092856 | Renal Cell Carcinoma |  | Recruiting | University of Southern California |
|  |  |  | NCT03971409 | Breast Cancer |  | Recruiting | University of California |
